# Supplementary material for: Iron atom–cluster interactions increase activity and improve durability in Fe–N–C fuel cells
Source: Nat Commun. 2022 May 26;13:2963. doi: 10.1038/s41467-022-30702-z (PMC9135695; doi:10.1038/s41467-022-30702-z)
Supplement: Supplementary file 1 — Supplementary Information [file 41467_2022_30702_MOESM1_ESM.pdf]

Supplementary Information for

Iron atom–cluster interactions increase activity and improve durability in Fe–N–C fuel cells

Xin Wan[1], Qingtao Liu[1], Jieyuan Liu[1], Shiyuan Liu[1], Xiaofang Liu[1], Lirong Zheng[2], Jiaxiang Shang[1], Ronghai Yu[1], and Jianglan Shui[1*]

[1]School of Materials Science and Engineering, Beihang University, Beijing 100191, China.

[2]Beijing Synchrotron Radiation Facility, Institute of High Energy Physics, Chinese Academy of Sciences, Beijing 100049, China.

*e-mail: shuijianglan@buaa.edu.cn

Contents:

Supplementary Figures 1 to 36

Supplementary Tables 1 to 8

Supplementary References

Supplymentary Figures

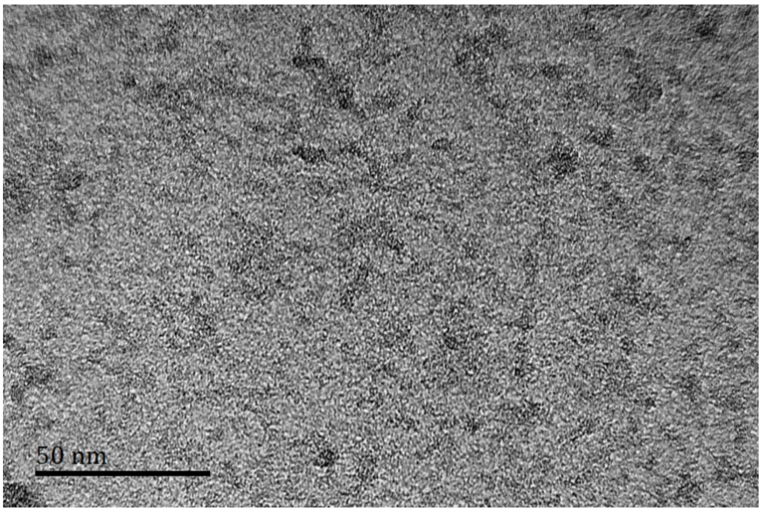

Supplementary Figure 1. TEM image of CQD made from ZIF-8.

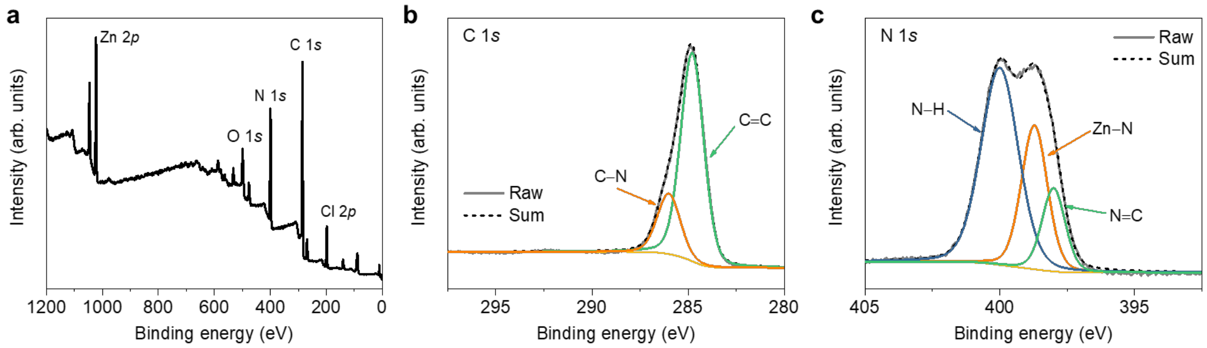

Supplementary Figure 2. XPS analysis of CQD. (**a**) XPS survey spectrum. (**b**) C 1*s* spectrum. (**c**) N 1*s* spectrum.

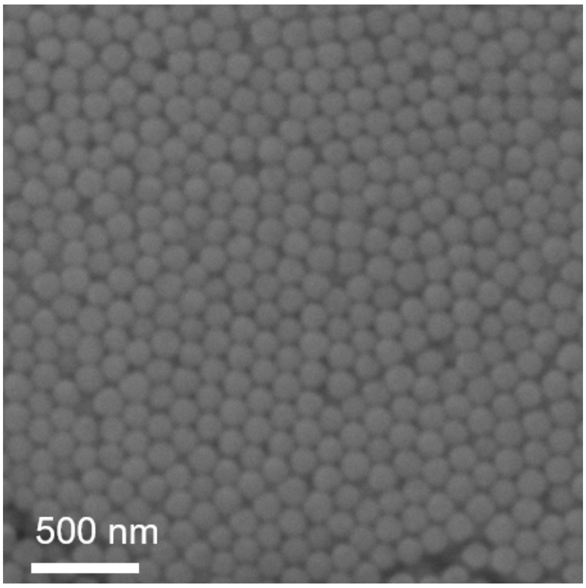

Supplementary Figure 3. SEM image of SiO_2 spheres.

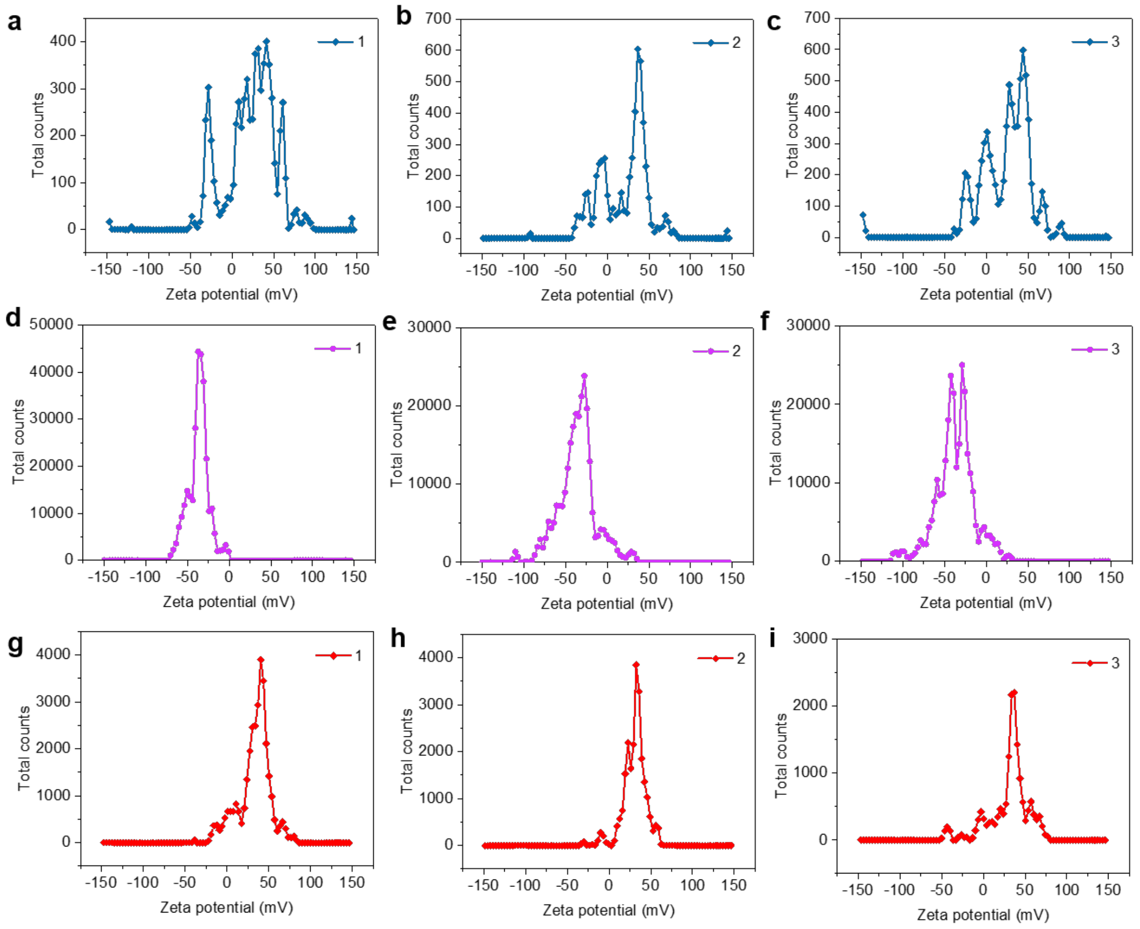

Supplementary Figure 4. Zeta potential of the precursors. (**a–c**) CQD; (**d–f**) SiO_2; (**g–i**) CQD-SiO_2-TPI mixture. Each sample was repeatedly measured three times.

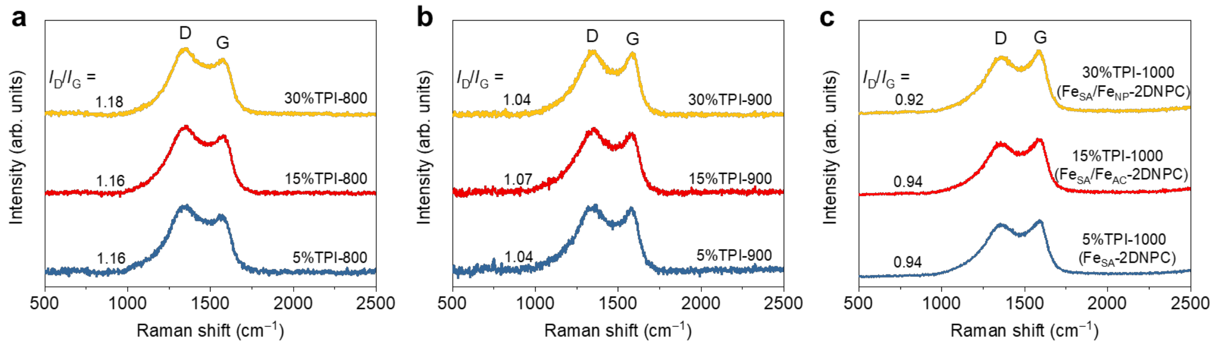

Supplementary Figure 5. Raman spectra of the catalysts. (**a**) *x*%-800; (**b**) *x*%-900; (**c**) *x*%-1000. The temperature of the second pyrolysis (T_2 /°C) was adjusted from 800 to 1000 °C. The samples were denoted as *x*%-T_2, where *x*% means the mass ratio of TPI relative to CQD in the precursor.

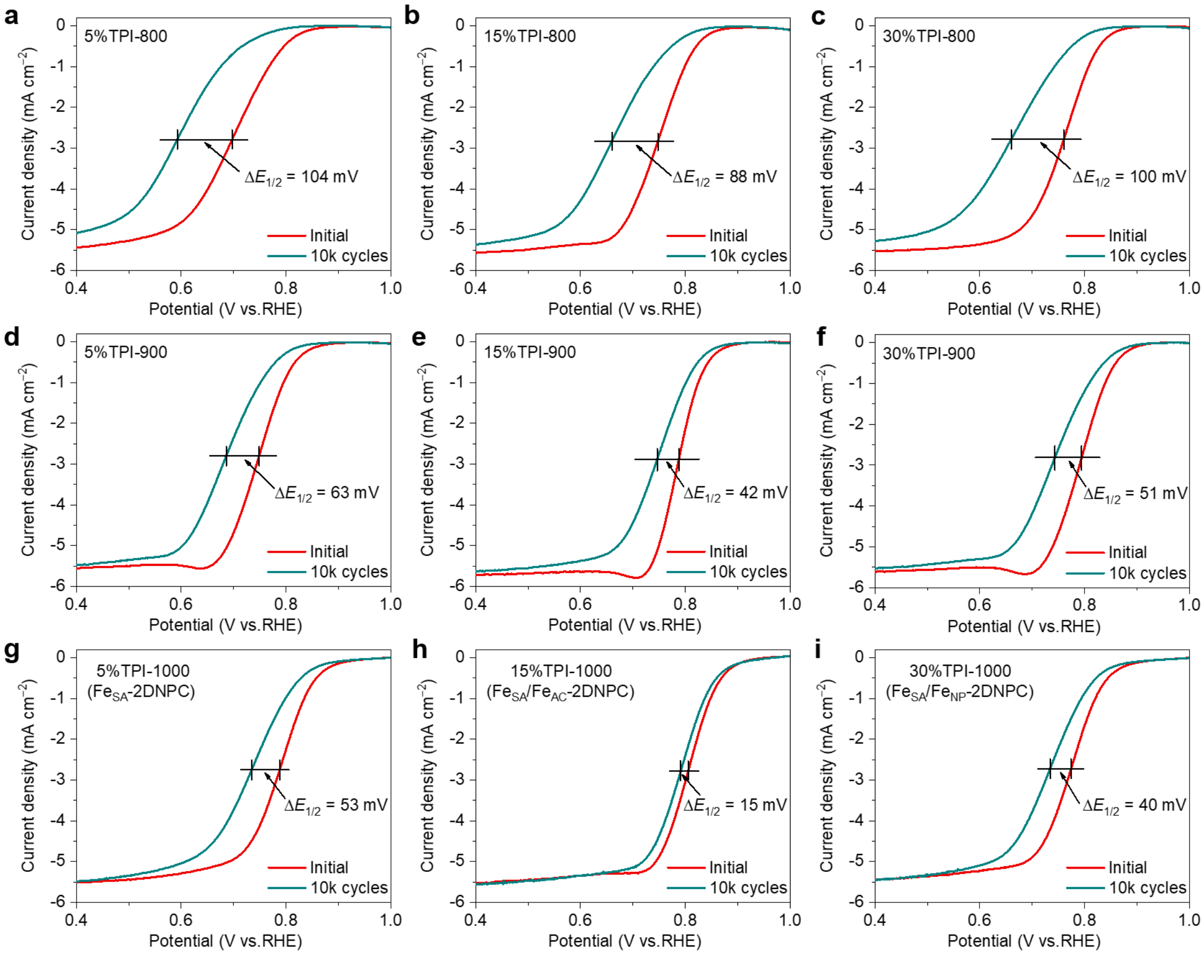

Supplementary Figure 6. Half-cell stability tests of x%-T_2 by potential cycling at room temperature. ORR polarization curves before and after 10,000 potential cycles (0.6–1.0 V vs. RHE) in O_2-purged 0.5 M H_2SO_4. (**a–c**) x%-800; (**d–f**) x%-900; (**g–i**) x%-1000.

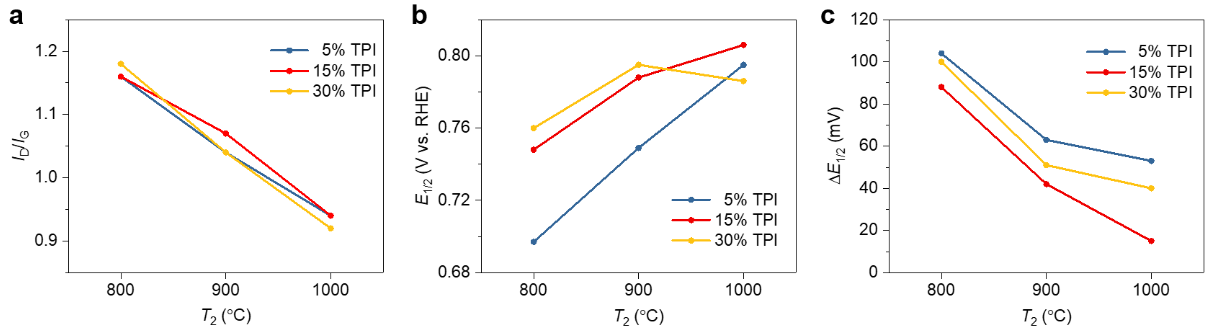

Supplementary Figure 7. Effect of the second pyrolysis temperature (T_2). Correlations between (**a**) graphitic degree (I_D/I_G), (**b**) ORR activity ($E_{1/2}$) and (**c**) stability ($\Delta E_{1/2}$) of the catalysts with T_2.

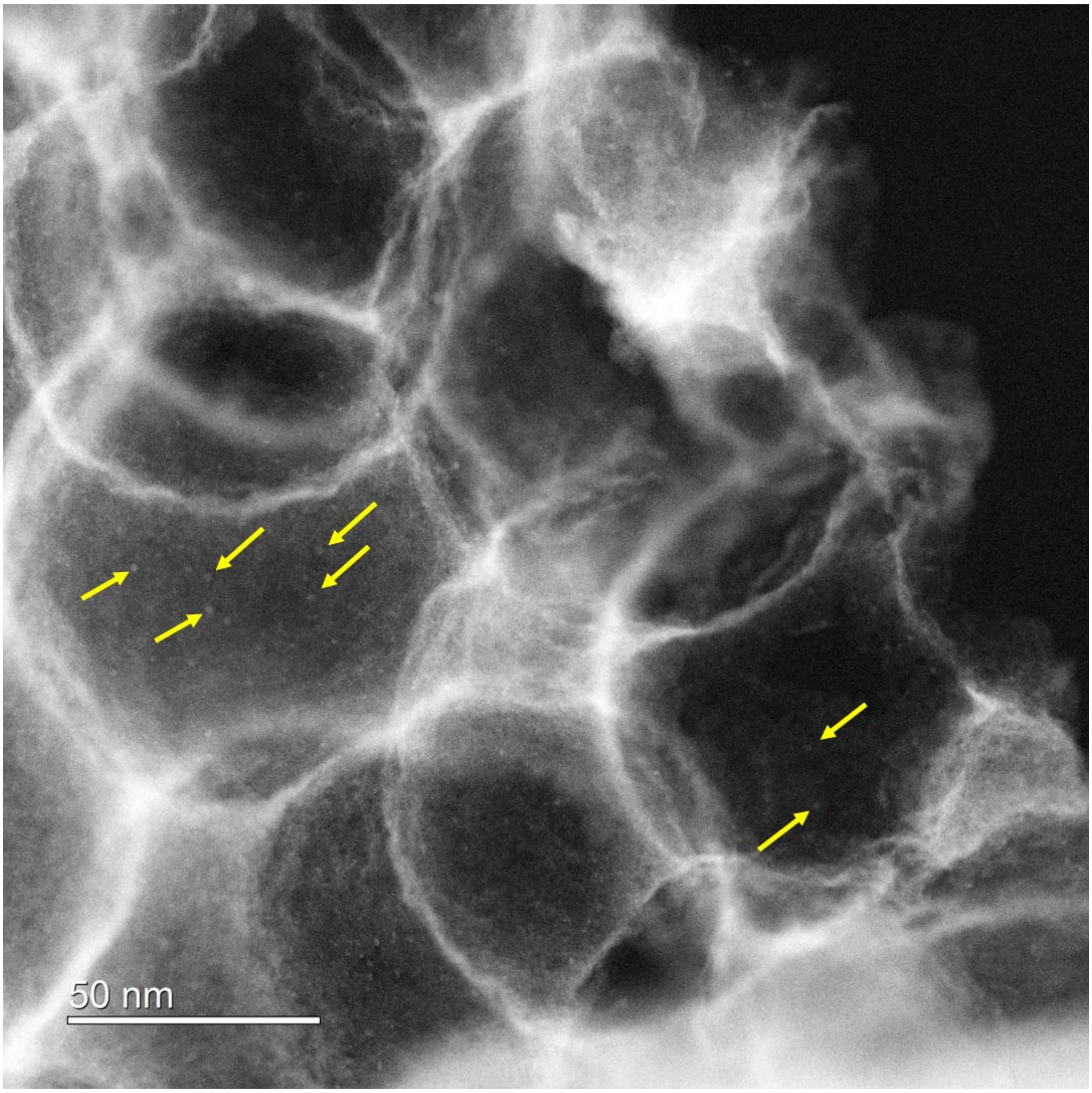

Supplementary Figure 8. HAADF-STEM image of Fe_{SA}/Fe_{AC}-2DNPC. The bright dots marked by the yellow arrows represent Fe ACs.

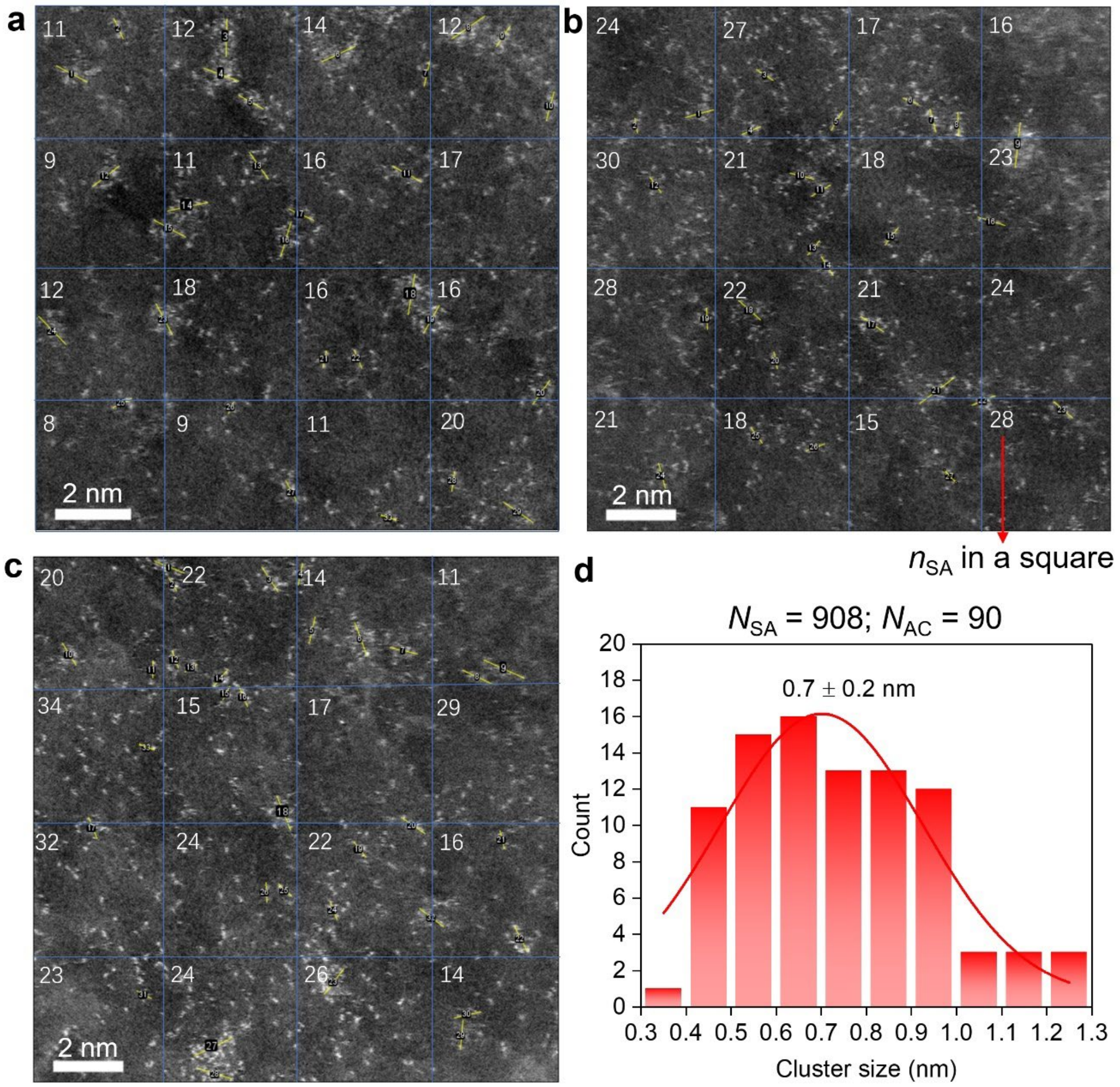

Supplementary Figure 9. Statistics of cluster size and SA-to-AC ratio in Fe_{SA}/Fe_{AC}-2DNPC. (**a–c**) Three different areas of HAADF-STEM images for statistics. The images are divided into small squares for counting single atoms. The number of single atoms in each square is marked in its upper left corner. (**d**) Size distribution histogram of the iron clusters in Fe_{SA}/Fe_{AC}-2DNPC.

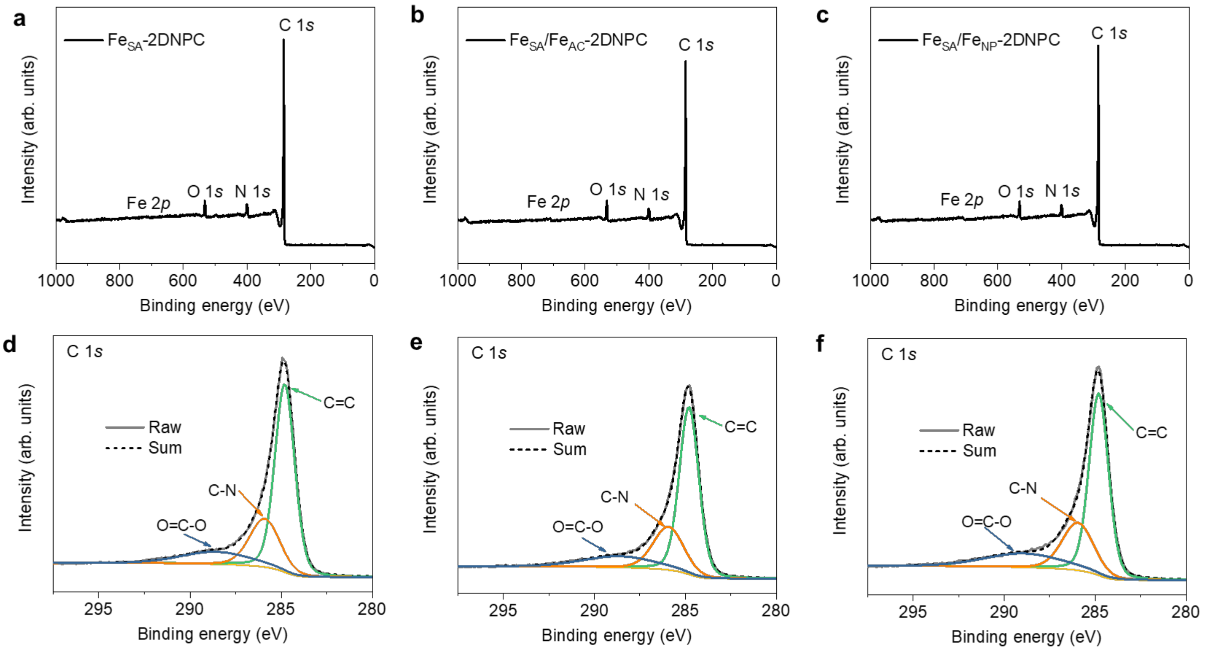

Supplementary Figure 10. XPS analyses of the catalysts. XPS survey spectra of (**a**) Fe$_{SA}$-2DNPC, (**b**) Fe$_{SA}$/Fe$_{AC}$-2DNPC and (**c**) Fe$_{SA}$/Fe$_{NP}$-2DNPC. High-resolution C 1*s* XPS spectra of (**d**) Fe$_{SA}$-2DNPC, (**e**) Fe$_{SA}$/Fe$_{AC}$-2DNPC and (**f**) Fe$_{SA}$/Fe$_{NP}$-2DNPC.

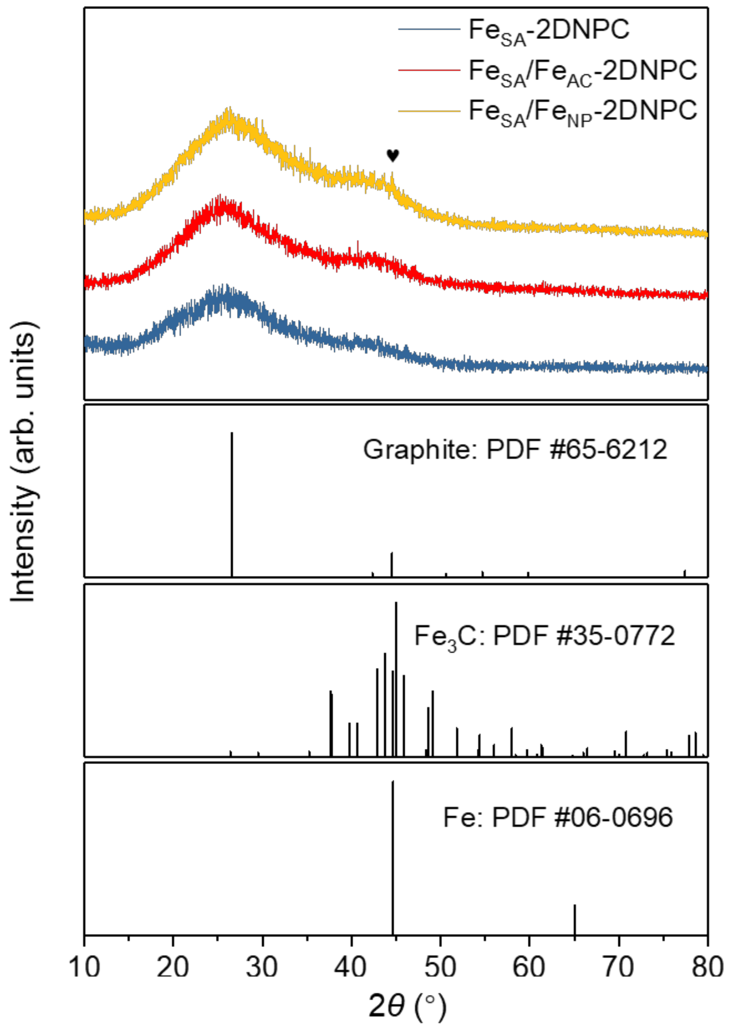

Supplementary Figure 11. XRD patterns of the catalysts. The weak signal of Fe-related peaks in the XRD pattern of Fe_{SA}/Fe_{NP}-2DNPC should be due to the small amount of Fe NPs.

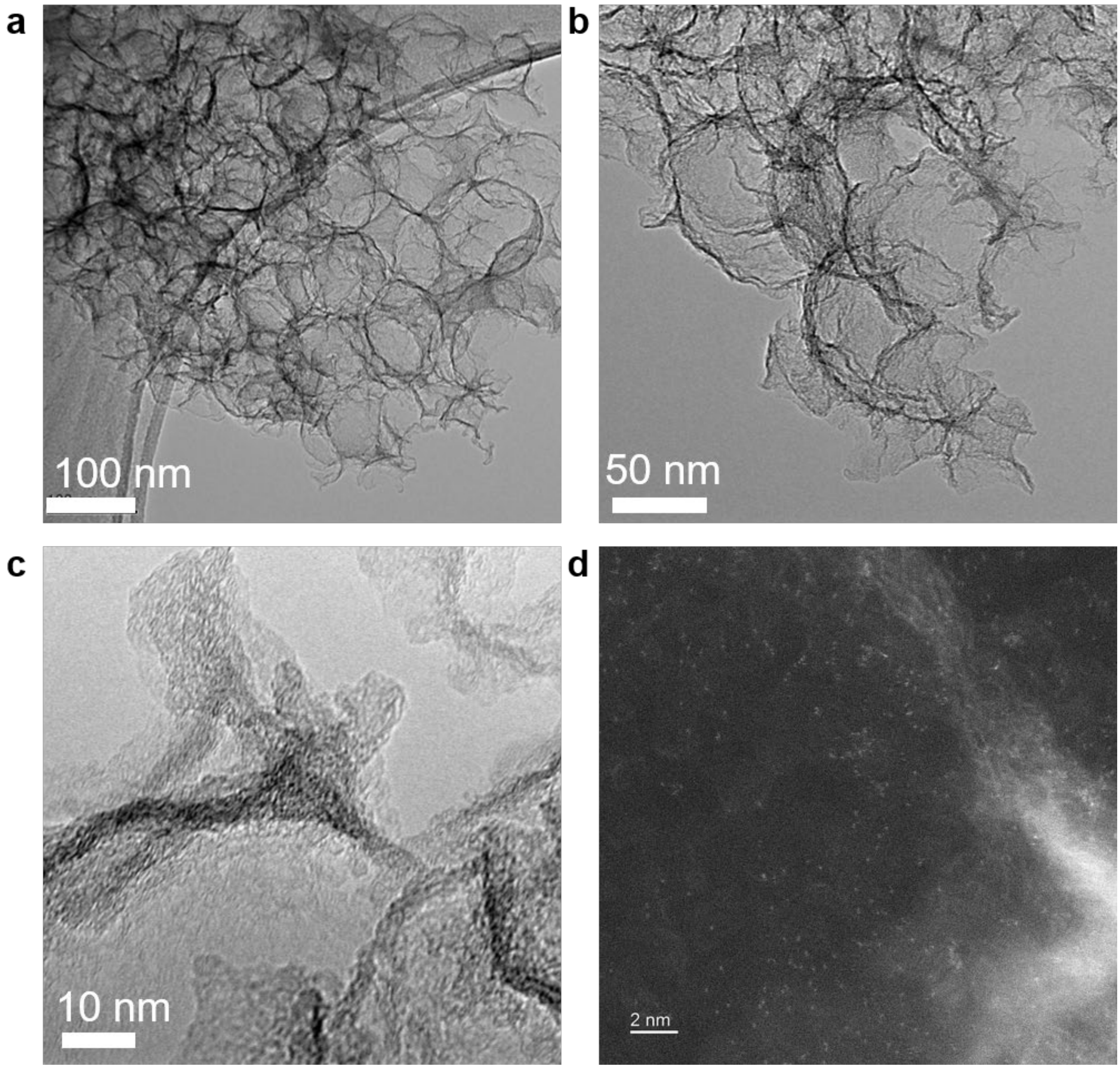

Supplementary Figure 12. Electron microscopic characterization of Fe_{SA}-2DNPC. (**a**–**c**) TEM and (**d**) HAADF-STEM images of Fe_{SA}-2DNPC.

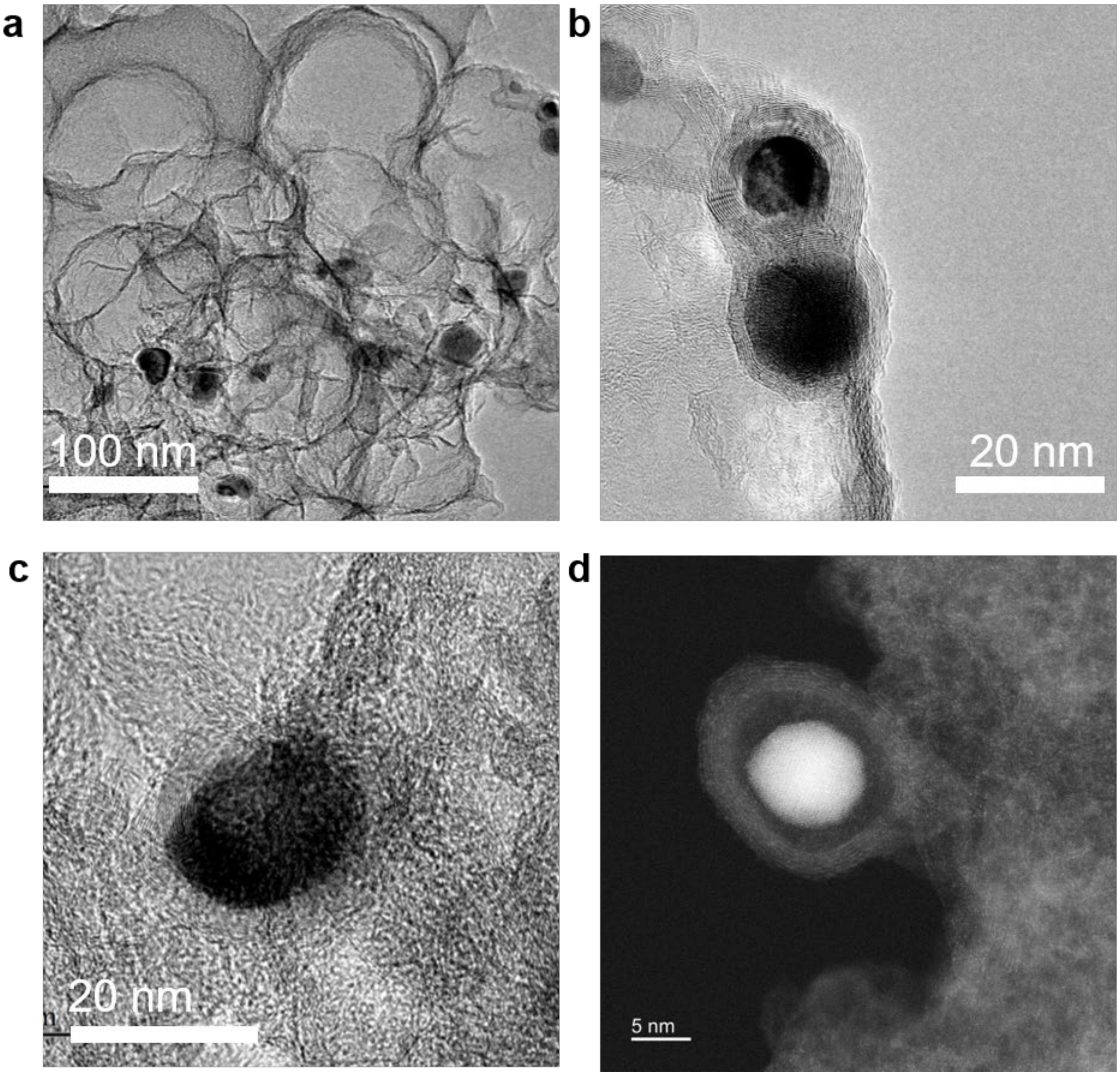

Supplementary Figure 13. Electron microscopic characterization of Fe_{SA}/Fe_{NP}-2DNPC. (**a–c**) TEM and (**d**) HAADF-STEM images of Fe_{SA}/Fe_{NP}-2DNPC.

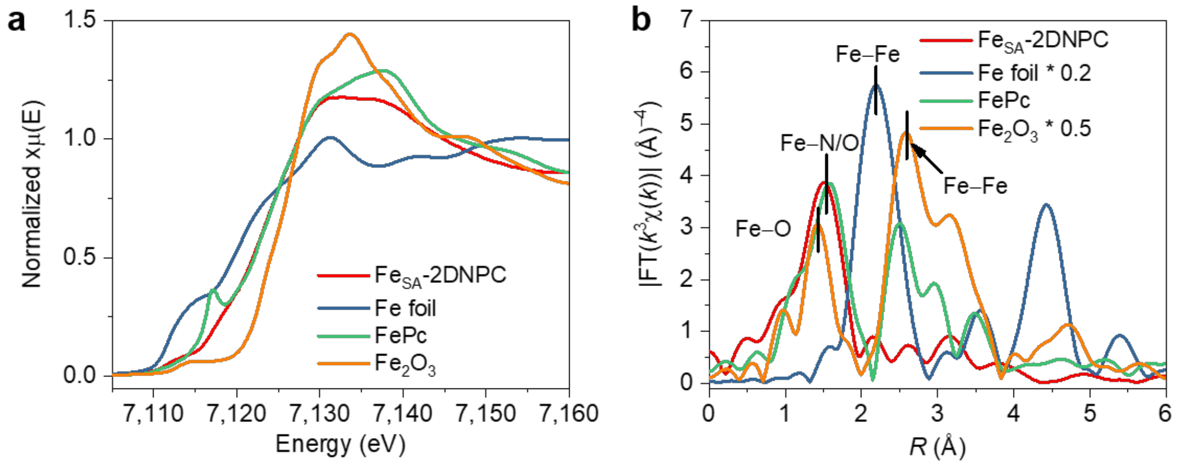

Supplementary Figure 14. XAS analysis of Fe$_{SA}$-2DNPC. (**a**) Normalized Fe K-edge XANES spectra; (**b**) k^3-weighted Fourier transforms of Fe$_{SA}$-2DNPC and references of Fe foil, FePc and Fe_2O_3.

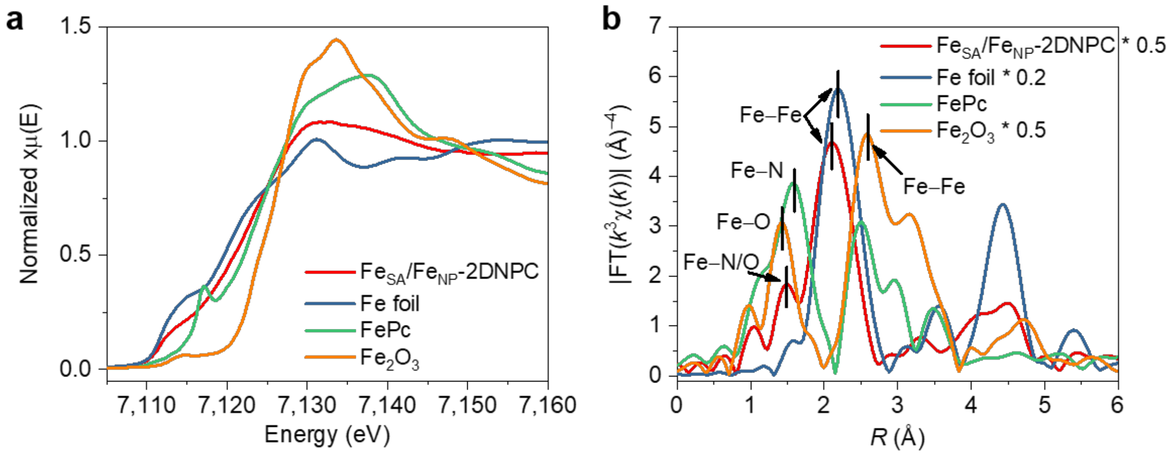

Supplementary Figure 15. XAS analysis of Fe$_{SA}$/Fe$_{NP}$-2DNPC. (**a**) Normalized Fe K-edge XANES spectra; (**b**) k^3-weighted Fourier transforms of Fe$_{SA}$/Fe$_{NP}$-2DNPC and references of Fe foil, FePc and Fe_2O_3.

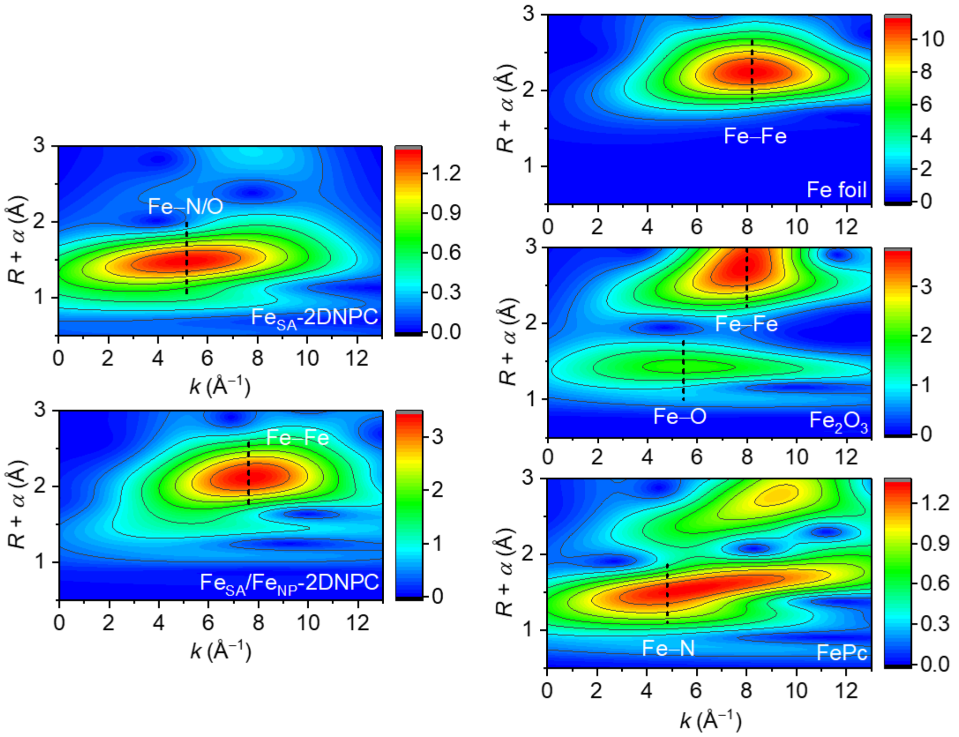

Supplementary Figure 16. EXAFS WT plots of the control samples. k^3-weighted wavelet transforms of the experimental EXAFS spectra of Fe_{SA}-2DNPC, Fe_{SA}/Fe_{NP}-2DNPC, and references of Fe foil, FePc and Fe_2O_3.

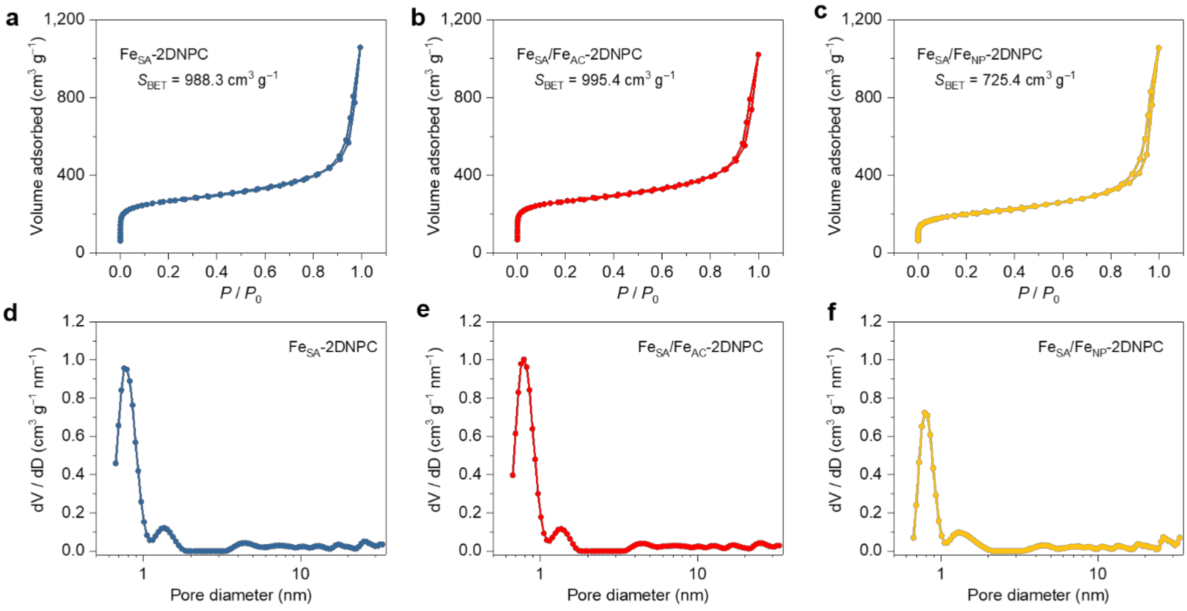

Supplementary Figure 17. Specific surface area and porosity analyses of the catalysts. (a–c) N_2 sorption isotherms and **(d–f)** pore size distributions. dV/dD, differential pore volume distribution; V, pore volume; D, pore diameter.

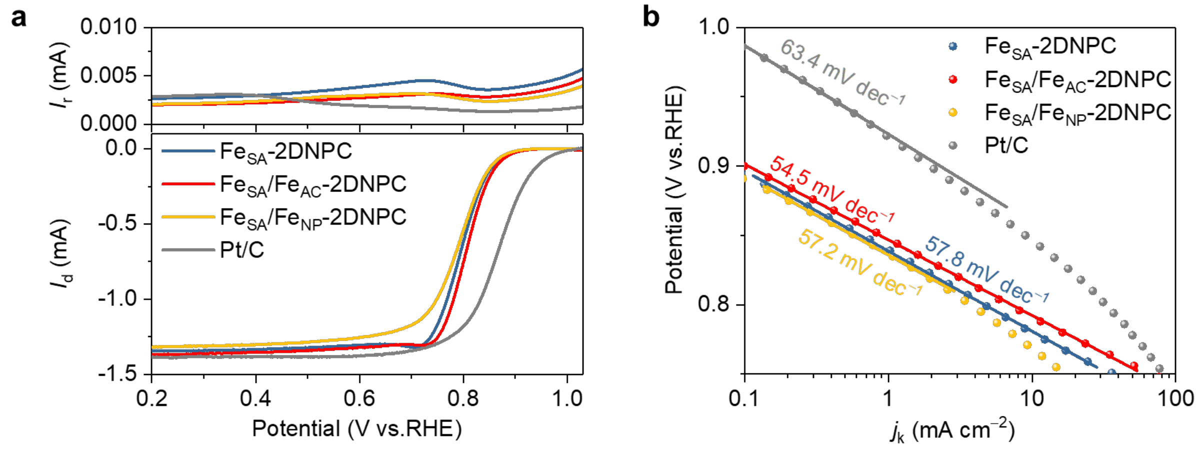

Supplementary Figure 18. The ring and disk current curves and corresponding Tafel plots. (**a**) The ring and disk current curves recorded on RRDE. (**b**) The corresponding Tafel plots of the indicated catalysts in Figure 3a,b.

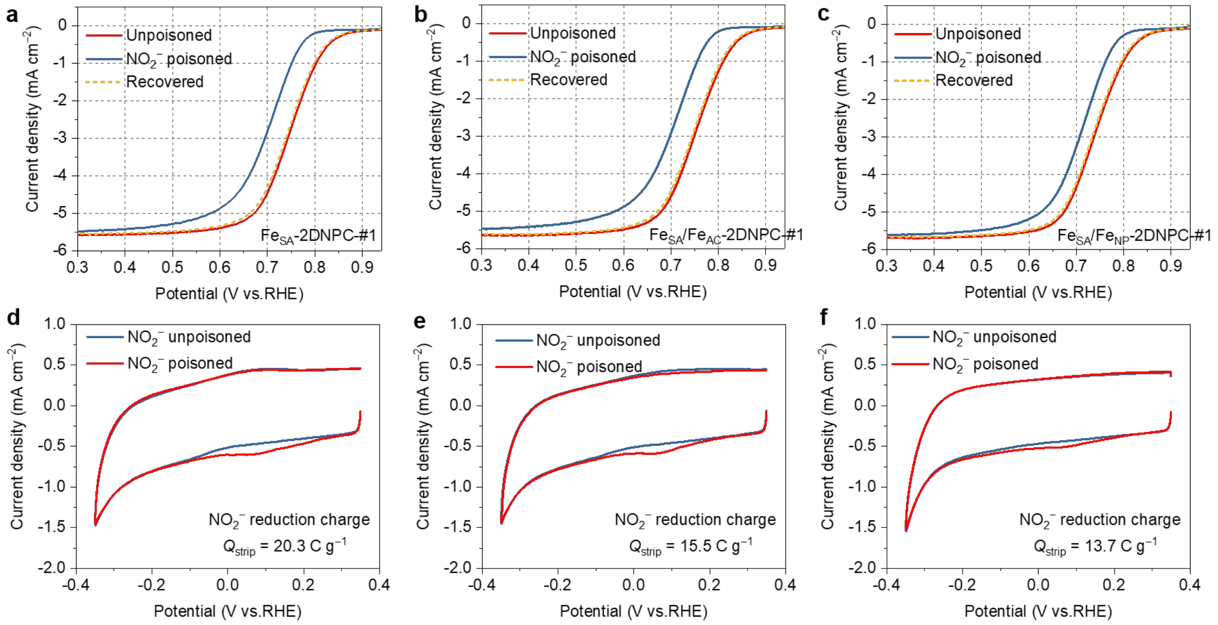

Supplementary Figure 19. Determination of SD and TOF of the catalysts by the method of nitrite adsorption and stripping. Batch #1. (**a–c**) LSV curves of the catalysts before and during nitrite adsorption, and after nitrite stripping in a 0.5 M acetate buffer at pH 5.2. (**d–f**) CV curves of catalysts before and during nitrite adsorption in the nitrite reductive stripping region. Catalyst loading, 0.15 mg cm^{-2}.

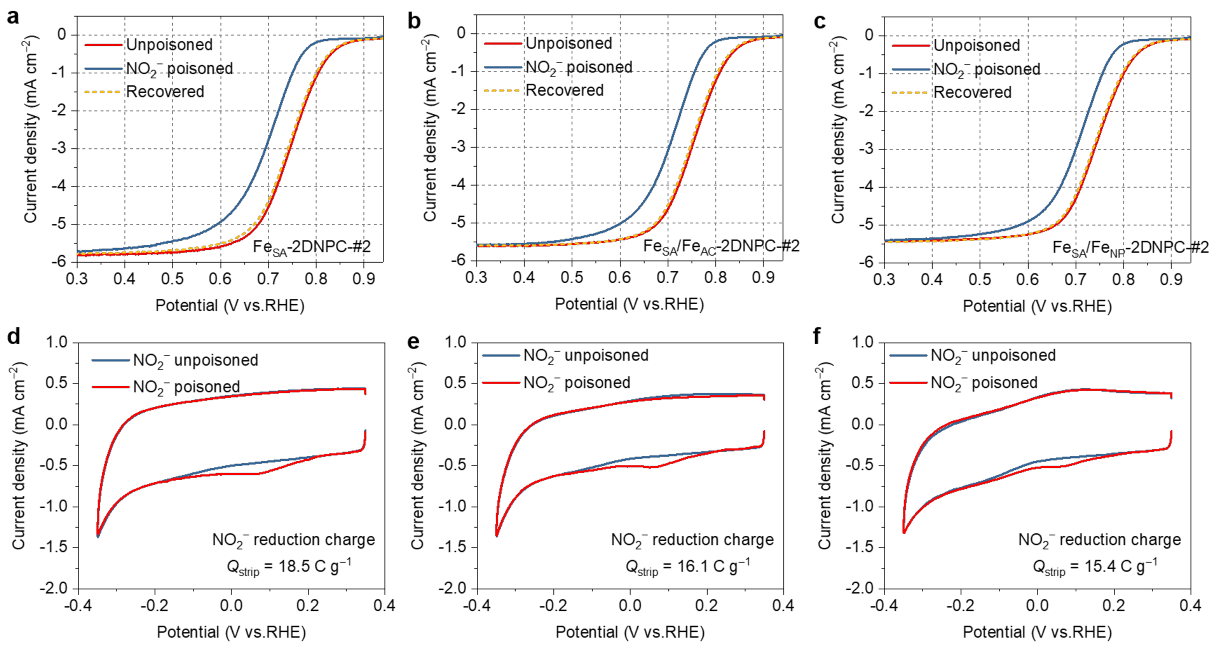

Supplementary Figure 20. Determination of SD and TOF of the catalysts by the method of nitrite adsorption and stripping. Batch #2. (**a–c**) LSV curves of the catalysts before and during nitrite adsorption, and after nitrite stripping in a 0.5 M acetate buffer at pH 5.2. (**d–f**) CV curves of catalysts before and during nitrite adsorption in the nitrite reductive stripping region. Catalyst loading, 0.15 mg cm^{-2}.

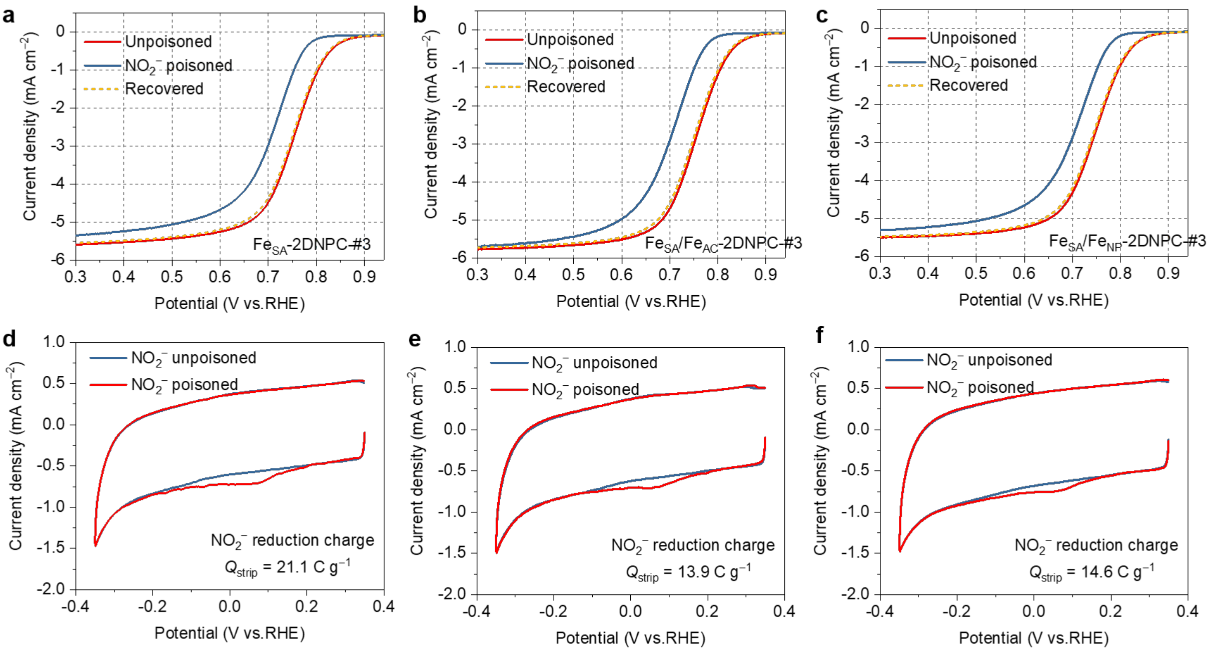

Supplementary Figure 21. Determination of SD and TOF of the catalysts by the method of nitrite adsorption and stripping. Batch #3. (**a–c**) LSV curves of the catalysts before and during nitrite adsorption, and after nitrite stripping in a 0.5 M acetate buffer at pH 5.2. (**d–f**) CV curves of catalysts before and during nitrite adsorption in the nitrite reductive stripping region. Catalyst loading, 0.15 mg cm^{-2}.

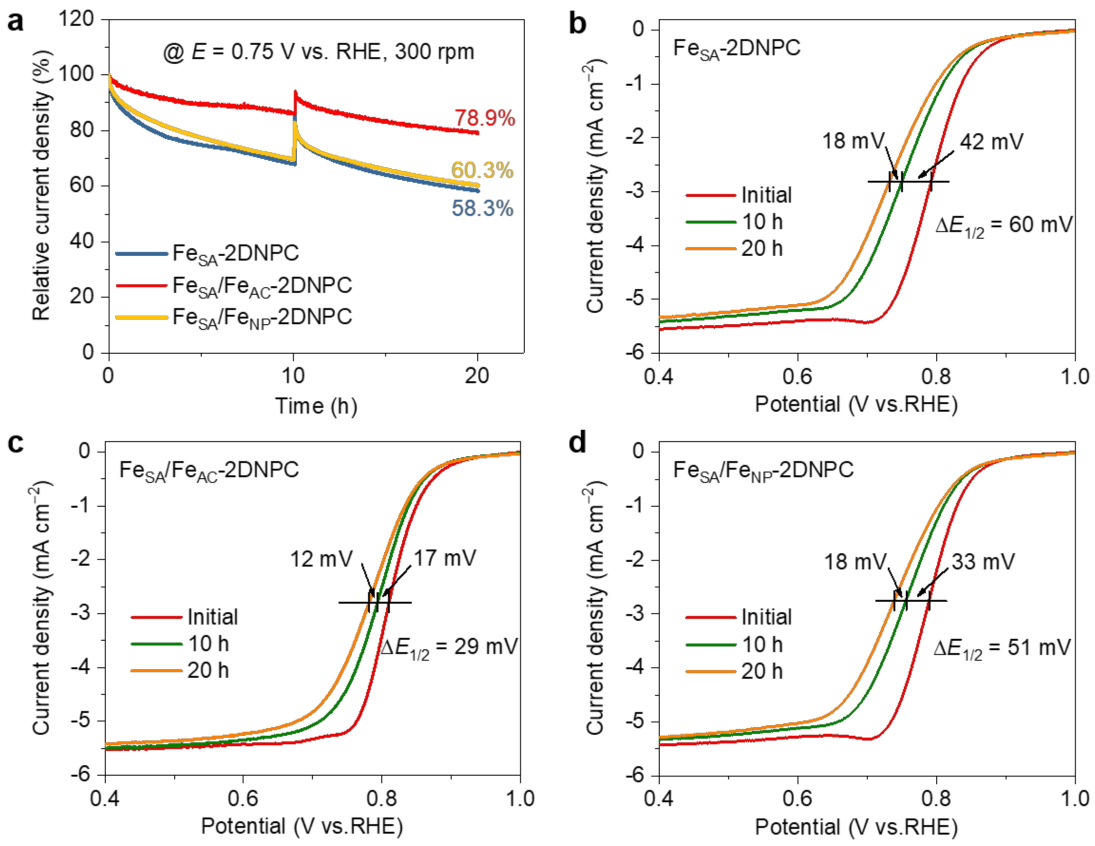

Supplementary Figure 22. Half-cell stability tests by chronoamperometry at room temperature. 20-h *i-t* tests at 0.75 V (**a**) for Fe_{SA}-2DNPC, Fe_{SA}/Fe_{AC}-2DNPC and Fe_{SA}/Fe_{NP}-2DNPC, during which the ORR polarization curves were recorded initially and every ten hours (**b–d**). Test conditions: O_2-purged 0.5 M H_2SO_4, 300 rpm, 25 °C; catalyst loading of 0.4 mg cm^{-2}, graphite rod as counter electrode. LSV curves were recorded at 1,600 rpm.

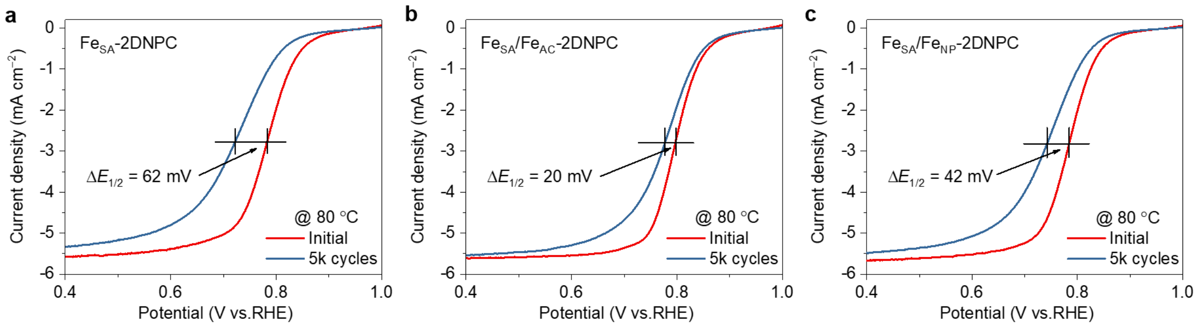

Supplementary Figure 23. Half-cell stability tests by potential cycling at 80 °C. ORR polarization curves before and after 5,000-cycle potential cycling for (**a**) Fe_{SA}-2DNPC, (**b**) Fe_{SA}/Fe_{AC}-2DNPC, and (**c**) Fe_{SA}/Fe_{NP}-2DNPC. Cycling conditions: 0.6–1.0 V vs. RHE, O_2-purged 0.5 M H_2SO_4, 50 mV s^{-1}, 300 rpm, 80 °C; catalyst loading of 0.4 mg cm^{-2}, graphite rod as counter electrode.

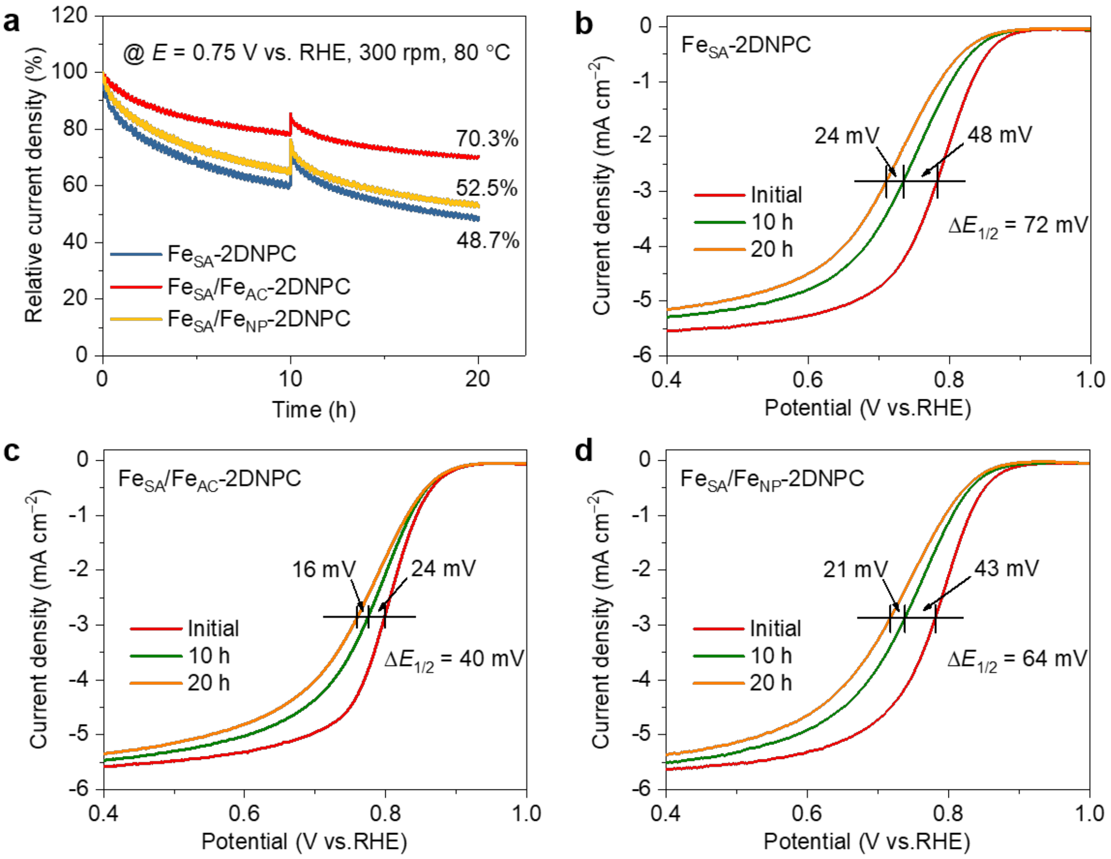

Supplementary Figure 24. Half-cell stability tests by chronoamperometry at 80 °C. 20-h *i-t* tests at 0.75 V (a) for Fe_{SA}-2DNPC, Fe_{SA}/Fe_{AC}-2DNPC and Fe_{SA}/Fe_{NP}-2DNPC, during which the polarization curves were record initially and every ten hours (b,c,d). Test conditions: O_2-purged 0.5 M H_2SO_4, 300 rpm, 80 °C; catalyst loading of 0.4 mg cm^{-2}, graphite rod as counter electrode. LSV curves were recorded at 1,600 rpm.

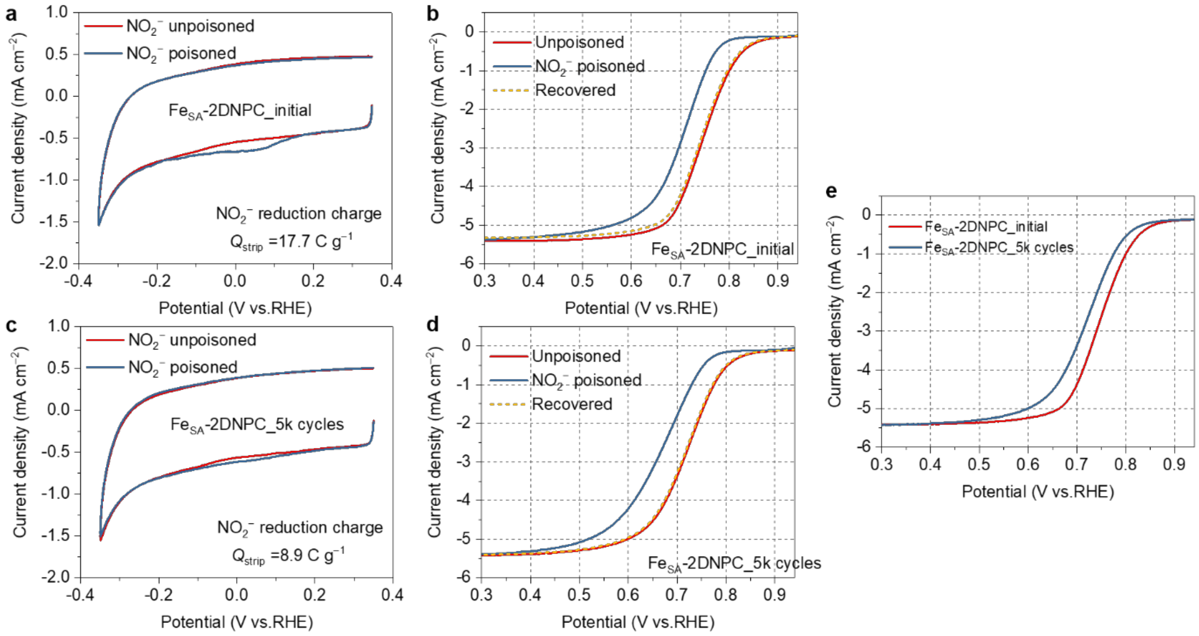

Supplementary Figure 25. Monitoring the changes of SD and TOF of Fe_{SA}-2DNPC before and after 5k CV cycles by the method of nitrite adsorption and stripping. (**a**) CV curves before and during nitrite adsorption in the nitrite reductive stripping region of fresh Fe_{SA}-2DNPC. (**b**) LSV curves before and during nitrite adsorption, and after nitrite stripping in a 0.5 M acetate buffer at pH 5.2 of fresh Fe_{SA}-2DNPC. (**c**) CV curves before and during nitrite adsorption in the nitrite reductive stripping region of the used Fe_{SA}-2DNPC after 5,000 potential cycles. (**d**) LSV curves before and during nitrite adsorption, and after nitrite stripping in a 0.5 M acetate buffer at pH 5.2 of the used Fe_{SA}-2DNPC after 5,000 potential cycles. (**e**) Comparison of the LSV curves of Fe_{SA}-2DNPC before and after 5,000 potential cycles. Cycling conditions: 0.6–1.0 V vs. RHE, O_2-purged 0.5 M H_2SO_4, 50 mV s^{-1}, 300 rpm, 25 °C; catalyst loading of 0.15 mg cm^{-2}, graphite rod as counter electrode.

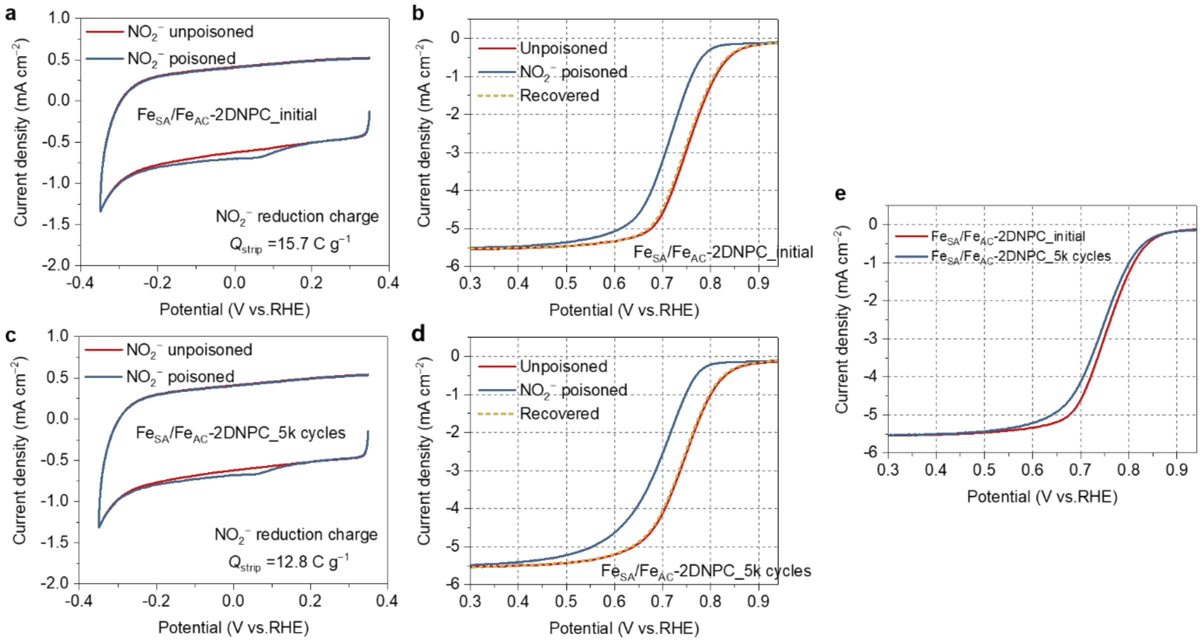

Supplementary Figure 26. Monitoring the changes of SD and TOF of Fe_{SA}/Fe_{AC}-2DNPC before and after 5k CV cycles by the method of nitrite adsorption and stripping. (**a**) CV curves before and during nitrite adsorption in the nitrite reductive stripping region of fresh Fe_{SA}/Fe_{AC}-2DNPC. (**b**) LSV curves before and during nitrite adsorption, and after nitrite stripping in a 0.5 M acetate buffer at pH 5.2 of fresh Fe_{SA}/Fe_{AC}-2DNPC. (**c**) CV curves before and during nitrite adsorption in the nitrite reductive stripping region of the used Fe_{SA}/Fe_{AC}-2DNPC after 5,000 potential cycles. (**d**) LSV curves before and during nitrite adsorption, and after nitrite stripping in a 0.5 M acetate buffer at pH 5.2 of the used Fe_{SA}/Fe_{AC}-2DNPC after 5,000 potential cycles. (**e**) Comparison of the LSV curves of Fe_{SA}/Fe_{AC}-2DNPC before and after 5,000 potential cycles.Cycling conditions: 0.6–1.0 V vs. RHE, O_2-purged 0.5 M H_2SO_4, 50 mV s^{-1}, 300 rpm, 25 °C; catalyst loading of 0.15 mg cm^{-2}, graphite rod as counter electrode.

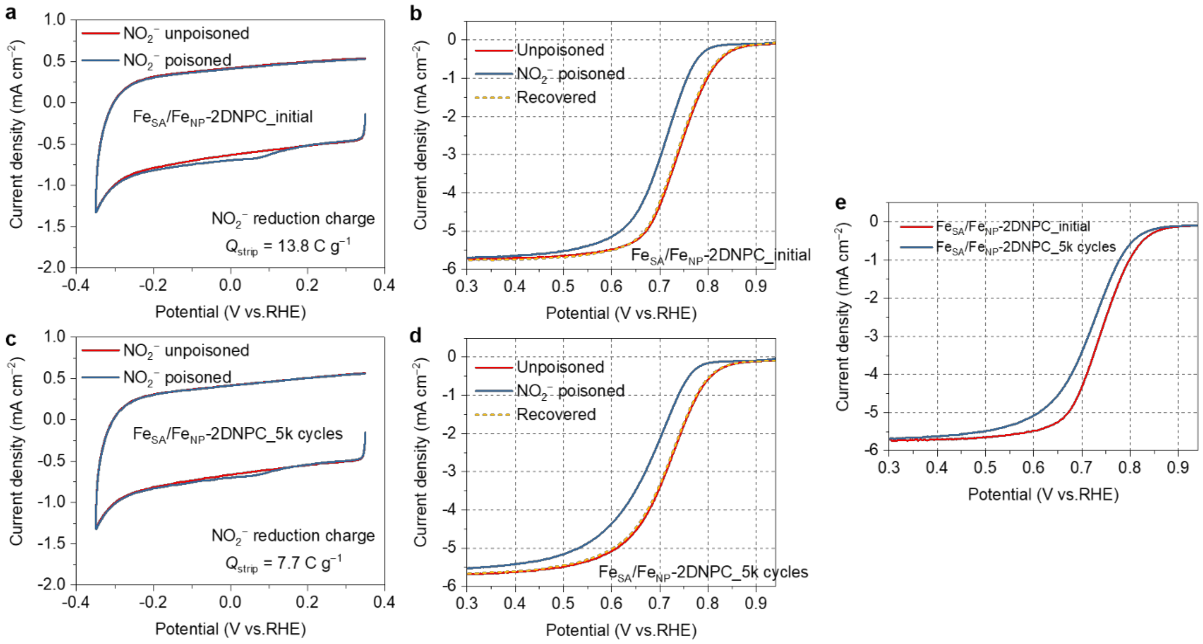

Supplementary Figure 27. Monitoring the changes of SD and TOF of Fe_{SA}/Fe_{NP}-2DNPC before and after 5k CV cycles by the method of nitrite adsorption and stripping. (**a**) CV curves before and during nitrite adsorption in the nitrite reductive stripping region of fresh Fe_{SA}/Fe_{NP}-2DNPC. (**b**) LSV curves before and during nitrite adsorption, and after nitrite stripping in a 0.5 M acetate buffer at pH 5.2 of fresh Fe_{SA}/Fe_{NP}-2DNPC. (**c**) CV curves before and during nitrite adsorption in the nitrite reductive stripping region of the used Fe_{SA}/Fe_{NP}-2DNPC after 5,000 potential cycles. (**d**) LSV curves before and during nitrite adsorption, and after nitrite stripping in a 0.5 M acetate buffer at pH 5.2 of the used Fe_{SA}/Fe_{NP}-2DNPC after 5,000 potential cycles. (**e**) Comparison of the LSV curves of Fe_{SA}/Fe_{NP}-2DNPC before and after 5,000 potential cycles. Cycling conditions: 0.6–1.0 V vs. RHE, O_2-purged 0.5 M H_2SO_4, 50 mV s^{-1}, 300 rpm, 25 °C; catalyst loading of 0.15 mg cm^{-2}, graphite rod as counter electrode.

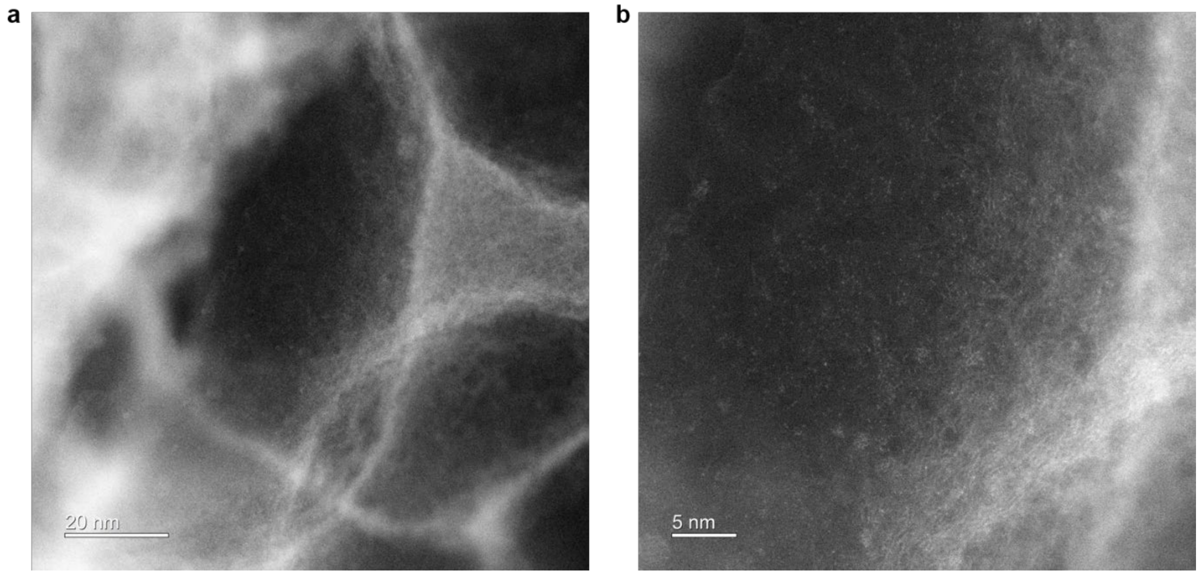

Supplementary Figure 28. HAADF-STEM images of the used Fe_{SA}/Fe_{AC}-2DNPC after 5,000 potential cycles. (**a**) HAADF-STEM image. (**b**) Atomic-resolution HAADF-STEM image. Cycling conditions: 0.6–1.0 V vs. RHE, O_2-purged 0.5 M H_2SO_4, 50 mV s^{-1}, 300 rpm, 25 °C; catalyst loading of 0.15 mg cm^{-2}, graphite rod as counter electrode.

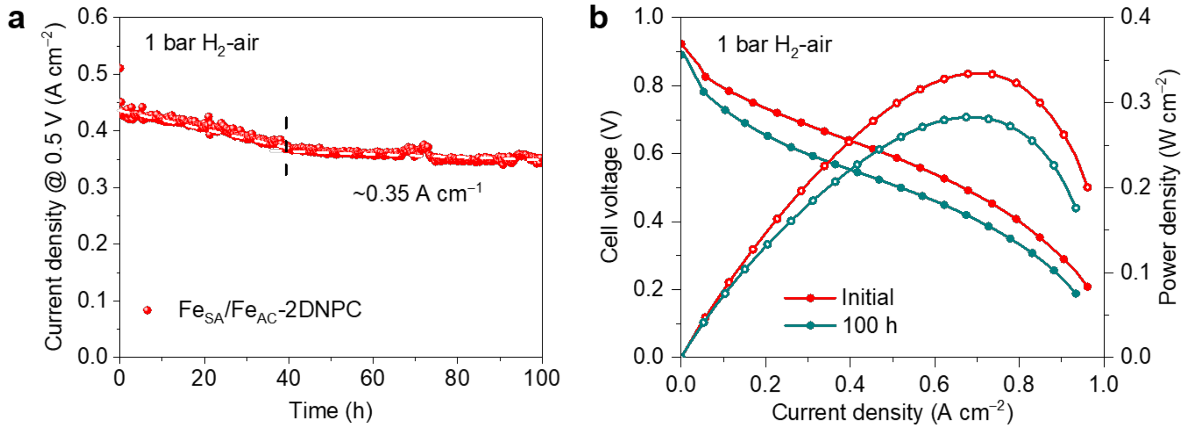

Supplementary Figure 29. Repeat stability test of Fe_{SA}/Fe_{AC}-2DNPC in PEMFC under 1 bar H_2–air. (**a**) The 100-h stability test of Fe_{SA}/Fe_{AC}-2DNPC at cell voltage of 0.5 V under 1 bar H_2–air. (**b**) Polarization and power density curves of Fe_{SA}/Fe_{AC}-2DNPC recorded initially and after 100 h.

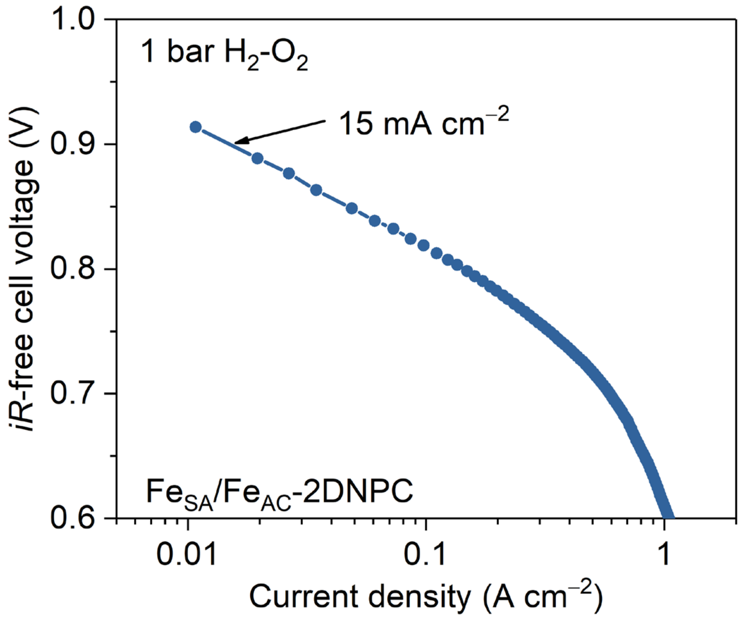

Supplementary Figure 30. Tafel plot for determination of the activity of Fe_{SA}/Fe_{AC}-2DNPC.

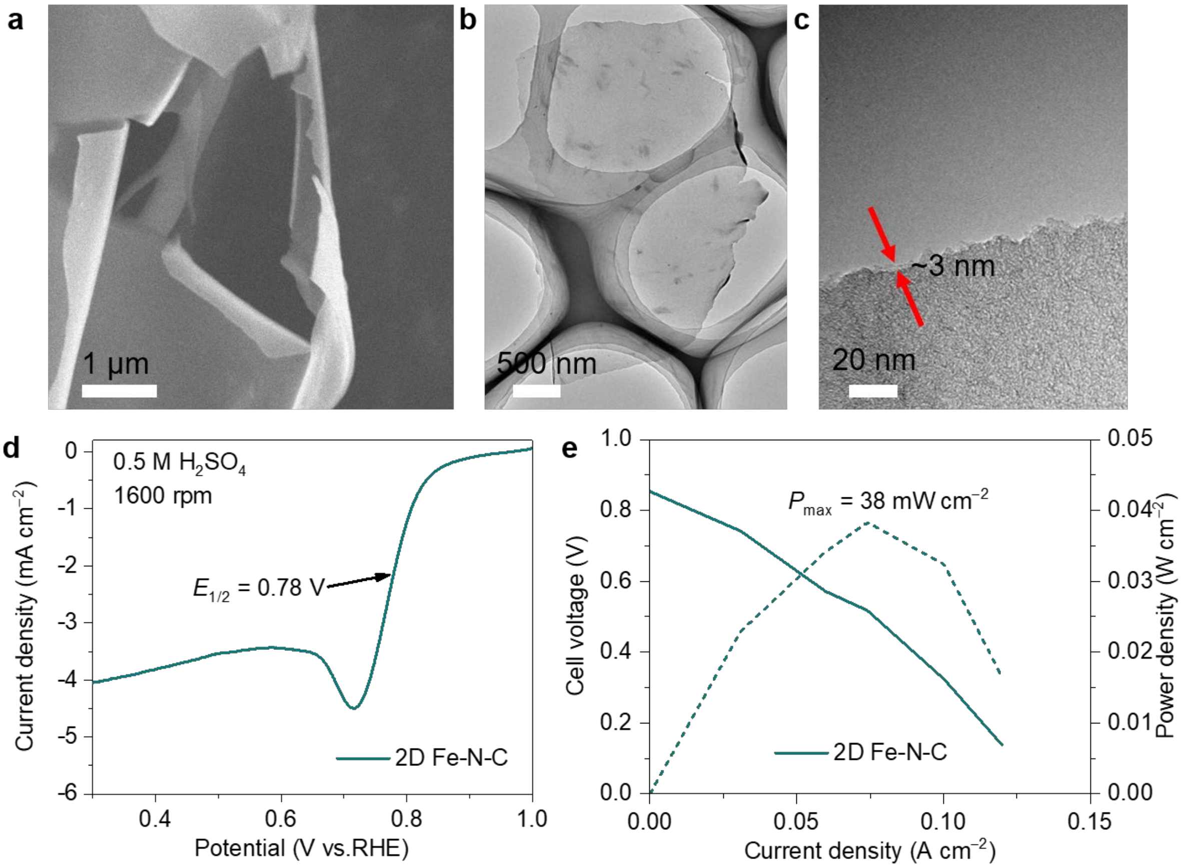

Supplementary Figure 31. Effect of SiO_2 template. (**a**) SEM image, (**b,c**) TEM images, (**d**) ORR polarization curve and (**e**) PEMFC polarization and power density curves of the control sample made without silica templates (2D Fe-N-C) under 1 bar H_2–O_2.

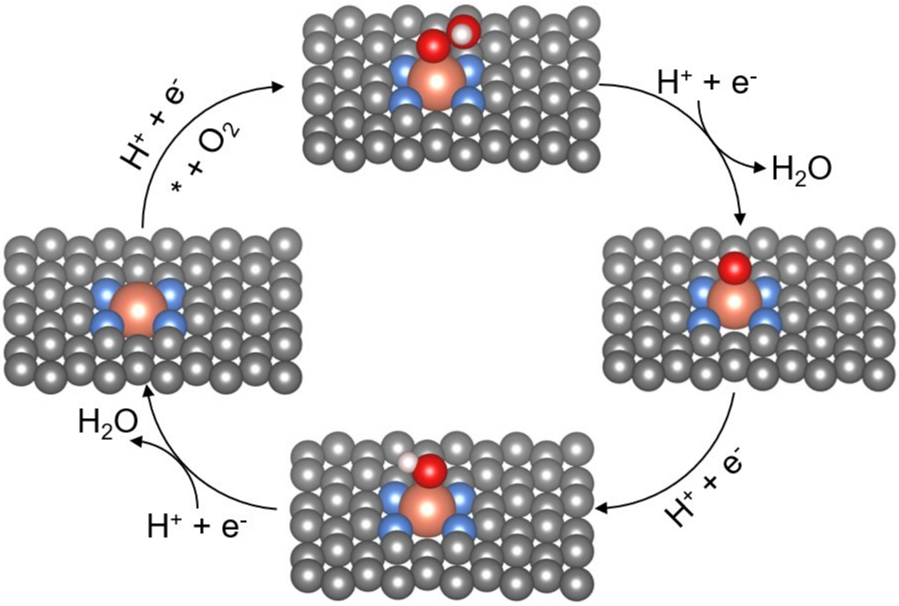

Supplementary Figure 32. Proposed ORR mechanism on Fe–N$_4$ site. C, gray; Fe, bronze; H, white; N, blue; O, red.

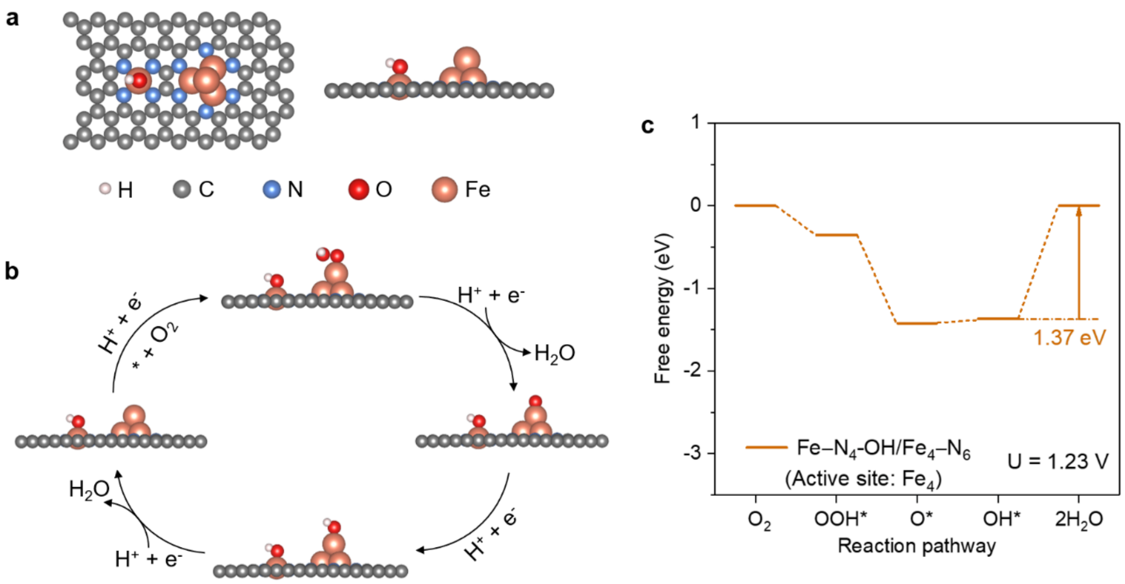

Supplementary Figure 33. Theoretical analysis of the activity of the iron cluster (Fe_4–N_6) in the hybrid active site. (**a**) Model structure of Fe–N_4-OH/Fe_4–N_6 used for theoretical calculation. (**b**) Schematic ORR process on the Fe_4 site of Fe–N_4-OH/Fe_4–N_6. (**c**) Free energy diagram at 1.23 V for ORR over the Fe_4 site of Fe–N_4-OH/Fe_4–N_6.

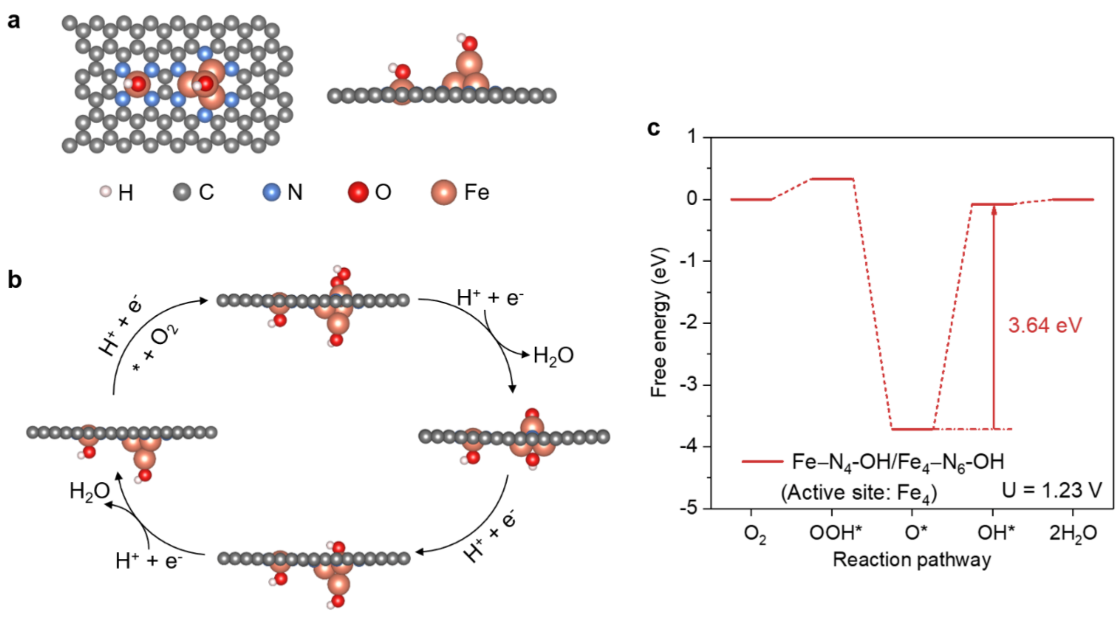

Supplementary Figure 34. Theoretical analysis of the activity of the OH-modified iron cluster (Fe_4–N_6-OH) in the hybrid active site. (**a**) Model structure of Fe–N_4-OH/Fe_4–N_6-OH used for theoretical calculation. (**b**) Schematic ORR process on the Fe_4 site of Fe–N_4-OH/Fe_4–N_6-OH. (**c**) Free energy diagram at 1.23 V for ORR over the Fe_4 site of Fe–N_4-OH/Fe_4–N_6-OH.

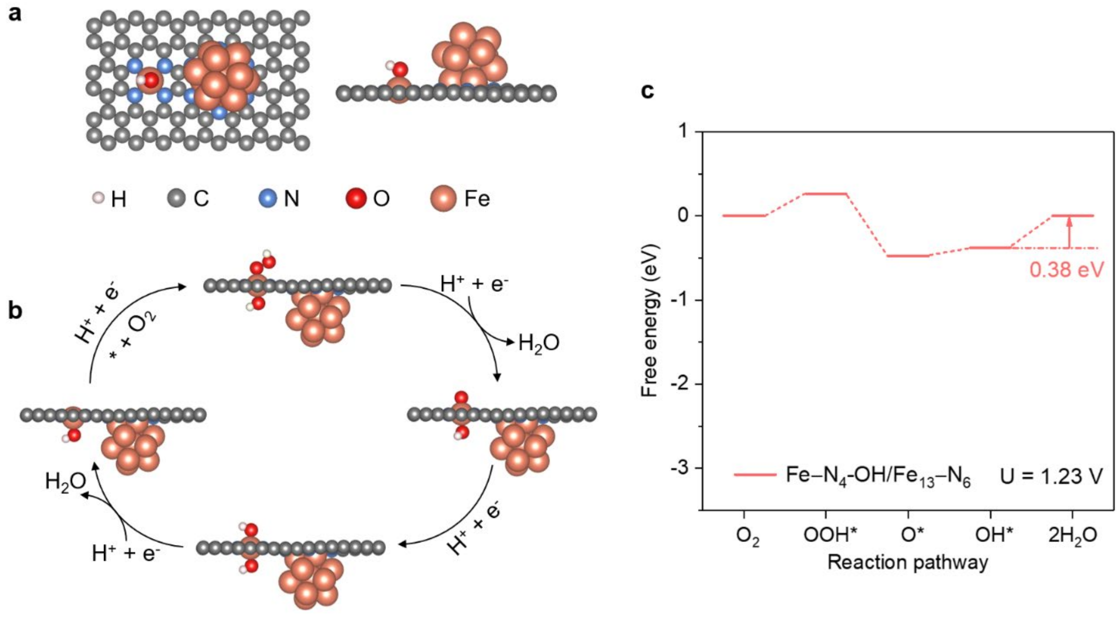

Supplementary Figure 35. Theoretical analysis of the activity of the hybrid active site with Fe_{13}–N_6 cluster. (**a**) Model structure of Fe–N_4/Fe_{13}–N_6 used for theoretical calculation with a spontaneously formed OH ligand. (**b)** Schematic ORR process on the Fe–N_4-OH site of Fe–N_4-OH/Fe_4–N_6. (**c)** Free energy diagram at 1.23 V for ORR over the Fe–N_4-OH site of Fe–N_4-OH/Fe_{13}–N_6.

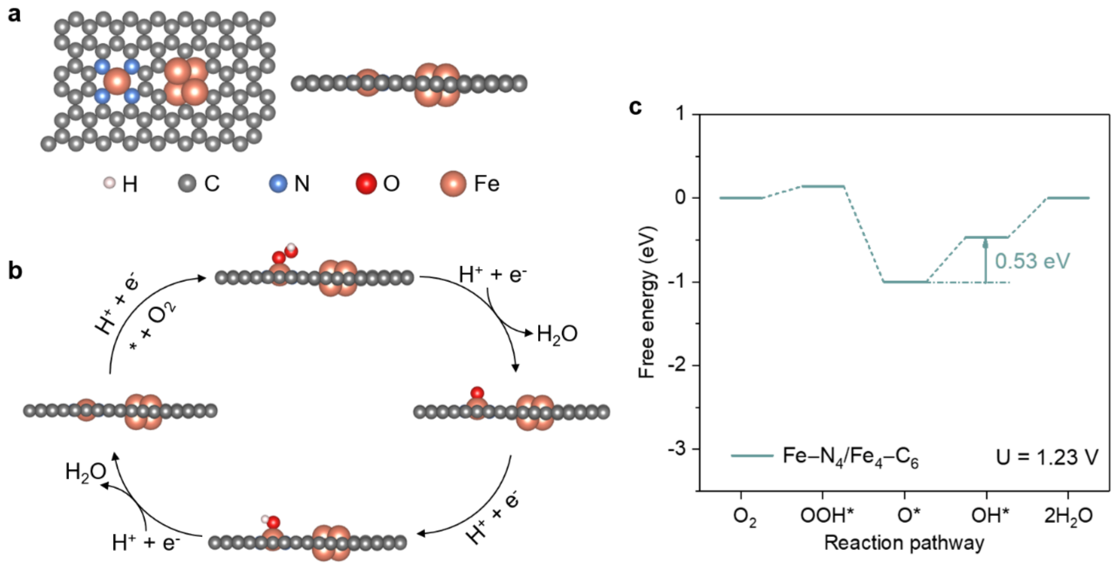

Supplementary Figure 36. Theoretical analysis of the activity of the hybrid active site with Fe_4–C_6 cluster. (**a**) Model structure of Fe–N_4/Fe_4–C_6 used for theoretical calculation. (**b)** Schematic ORR process on the Fe–N_4 site of Fe–N_4/Fe_4–C_6. (**c)** Free energy diagram at 1.23 V for ORR over the Fe–N_4 site of Fe–N_4/Fe_4–C_6.

Supplementary Tables

Supplementary Table 1. Fitting results of Fe K-edge EXAFS spectrum of Fe_{SA}/Fe_{AC}-2DNPC.

Sample	Path	***N***	***R* (Å)**	**σ^2 (Å^2)**	**ΔE_0 (eV)**	**S_0^2**	***R* factor (%)**
**Fe_{SA}/Fe_{AC}-2DNPC**	Fe–N/O	5.17	2.02	0.011	3.6	0.9	0.49
	Fe–Fe	0.38	2.55	0.006			
	Fe–Fe	0.33	3.05	0.006			

N is coordination number, *R* is the distance between absorber and backscatter atoms, σ^2 is Debye-Waller factor to account for both thermal and structural disorders, ΔE_0 is inner potential correction; *R* factor indicates the goodness of the fit. Error bounds (accuracies) that characterize the structural parameters obtained by EXAFS spectroscopy were estimated as $N \pm 20\%$; $R \pm 1\%$; $\sigma^2 \pm 20\%$; $\Delta E_0 \pm 20\%$. S_0^2 was fixed to 0.9 as determined from Fe foil fitting. Fitting range: $2.5 \leq k$ (/Å) ≤ 10.8 and $1 \leq R$ (Å) ≤ 3.

Supplementary Table 2. Surface elemental composition of catalysts determined by XPS.

	**C% (at/wt)**	**N% (at/wt)**	**O% (at/wt)**	**Fe% (at/wt)**
**Fe_{SA}-2DNPC**	89.20/86.38	6.35/7.17	4.22/5.45	0.22/0.99
**Fe_{SA}/Fe_{AC}-2DNPC**	87.17/83.74	6.69/7.50	5.86/7.51	0.28/1.26
**Fe_{SA}/Fe_{NP}-2DNPC**	88.49/84.94	6.16/6.90	4.94/6.32	0.41/1.83

Supplementary Table 3. Specific surface area and porosity of the catalysts.

	S_{BET} / m^2 g^{-1}	Micropore area / m^2 g^{-1}	External surface area / m^2 g^{-1}
**Fe_{SA}-2DNPC**	988.3	657.6	330.7
**Fe_{SA}/Fe_{AC}-2DNPC**	995.4	666.5	328.9
**Fe_{SA}/Fe_{NP}-2DNPC**	725.4	434.9	290.5

Supplementary Table 4. Summary of SD and TOF determined by the nitrite stripping experiments.

	**Batch No.**	**Q_{strip} (C g^{-1})**	**SD (μmol g^{-1})**	**J_{kin}-before @0.8V (mA cm^{-2})**	**J_{kin}-after @0.8V (mA cm^{-2})**	**TOF @0.8V (s^{-1})**
**Fe_{SA}-2DNPC**	1	20.3	42.1	1.145	0.101	1.70
	2	18.5	38.4	1.176	0.113	1.92
	3	21.1	43.7	1.222	0.107	1.76
**Fe_{SA}/Fe_{AC}-2DNPC**	1	15.5	32.1	1.468	0.135	2.87
	2	16.1	33.4	1.432	0.143	2.66
	3	13.9	28.8	1.323	0.099	2.94
**Fe_{SA}/Fe_{NP}-2DNPC**	1	13.7	28.4	0.990	0.205	1.91
	2	15.4	31.9	1.083	0.149	2.01
	3	14.6	30.3	1.022	0.106	2.10

Supplementary Table 5. Results of metal leaching experiments (before and after 5k CV cycles) by ICP.

		**Fe content (wt%)**	**5k_CV/initial**
**Fe_{SA}-2DNPC**	initial	0.59	64.4%
	5k_CV	0.38	
**Fe_{SA}/Fe_{AC}-2DNPC**	initial	1.16	84.5%
	5k_CV	0.98	
**Fe_{SA}/Fe_{NP}-2DNPC**	initial	1.56	78.8%
	5k_CV	1.23	

Supplementary Table 6. Summary of SD and TOF of the catalysts before and after 5k-CV stability tests.

		Q_{strip} (C g^{-1})	SD (μmol g^{-1})	J_{kin}-before @0.8V (mA cm^{-2})	J_{kin}-after @0.8V (mA cm^{-2})	TOF @0.8V (s^{-1})
**Fe_{SA}-2DNPC**	initial	17.7	36.7	1.054	0.111	1.78
	5k_CV	8.9	18.5	0.466	0.073	1.47
	initial/5k_CV		50.4%	44.2%		82.6%
**Fe_{SA}/Fe_{AC}-2DNPC**	initial	15.7	32.6	1.398	0.172	2.60
	5k_CV	12.8	26.5	1.011	0.092	2.39
	initial/5k_CV		81.3%	72.3%		91.9%
**Fe_{SA}/Fe_{NP}-2DNPC**	initial	13.8	28.6	1.006	0.155	2.06
	5k_CV	7.7	16.0	0.526	0.086	1.91
	initial/5k_CV		55.9%	52.3%		92.7%

Supplementary Table 7. Comparison of PEMFC stability of Fe_{SA}/Fe_{AC}-2DNPC with other reported M–N–C catalysts under 1 bar H_2–air.

Catalyst	Cell voltage (V)	Time (h)	Initial current density (mA cm^{-2})	Final current density (mA cm^{-2})	Decay rate (mA cm^{-2} h^{-1})	Decay rate (% h^{-1})	Ref. No.
**Fe_{SA}/Fe_{AC}-2DNPC**	0.5	150	440	365	0.5	0.11	This work
**d-(Co_{NP}/Co_{SA}–N–C)**	0.6	100	185	90	0.95	0.51	1
**P(AA-MA)(5-1)-Fe-N**	0.55	40	81	73	0.2	0.25	2
**Co(mIm)–NC(1.0)**	0.7	100	125	100	0.25	0.2	3
**FeN_4/HOPC-c-1000**	0.55	100	638	395	2.43	0.38	4
**FeN_x/GM**	0.4	93	449	253	2.1	0.47	5
**1.5Fe–ZIF**	0.55	96	543	291	2.6	0.48	6
**TPI@Z8(SiO_2)-650-C**	0.5	20	365	222	7.15	1.95	7
**20Mn-NC-second**	0.7	120	28	17	0.09	0.33	8
**20Co-NC-1100**	0.7	100	40	15	0.25	0.63	9
**Fe/N/CF**	0.5*	100	350	209	1.41	0.4	10
**1/20/80-Z8-1050 °C-15 min**	0.5*	100	593	284	3.1	0.52	11
**1/20/80-Z8-1050 °C**	0.5*	100	303	284	0.19	0.06	
**PANI-FeCo-C(1)**	0.4	700	347	337	0.014	0.004	12

*2 bar H_2–air

Supplementary Table 8. Comparison of mass activity of Pt-group-metal free catalysts in PEMFC under 1 bar H_2–O_2.

**Catalyst**	**Current density @ 0.9V$_{iR\text{-}free}$ (A cm^{-2})**	**Loading (mg cm^{-2})**	**Mass activity @0.9V$_{iR\text{-}free}$ (mA mg$_{cat.}^{-1}$)**	**Ref. No.**
**Fe$_{SA}$/Fe$_{AC}$-2DNPC**	0.015	1.5	10	This work
**FeNC-CVD-750**	0.033	6	5.5	13
**Co(mIm)–NC(1.0)**	0.022	6.3	3.5	3
**NDC-Fe-HT**	0.0045	2	2.3	14
**ZIF-NC-0.5Fe-700**	0.030	3.5	8.6	15
**TPI@Z8(SiO_2)-650-C**	0.022	2.7	8.1	7
**Fe2-Z8-C**	0.014*	1.9	7.4	16
**(CM+PANI)-Fe-C**	0.016	4	4	17
**Zn(eIm)rho-950**	0.004*	1	4	18
**Fe/N/CF**	0.010	2	5	10
**Zn(eIm)$_2$TPIP**	0.015*	2.2	6.8	19

*These values are not directly given in the papers, thus obtained by digging the polarization curves or calculated by the data provided.

Supplementary References

1. Cheng, X. *et al.* Nano-geometric deformation and synergistic Co nanoparticles—Co-N_4 composite sites for proton exchange membrane fuel cells. *Energy Environ. Sci.* **14**, 5958–5967 (2021).
2. Miao, Z. *et al.* Improving the stability of non-noble-metal M–N–C catalysts for proton-exchange-membrane fuel cells through M–N bond length and coordination regulation. *Adv. Mater.* **33**, 2006613 (2021).
3. Xie, X. *et al.* Performance enhancement and degradation mechanism identification of a single-atom Co–N–C catalyst for proton exchange membrane fuel cells. *Nat. Catal.* **3**, 1044–1054 (2020).
4. Qiao, M. *et al.* Hierarchically ordered porous carbon with atomically dispersed FeN_4 for ultra-efficient oxygen reduction reaction in proton-exchange membrane fuel cells. *Angew. Chem. Int. Ed.* **59**, 2688–2694 (2020).
5. Fu, X. *et al.* Tailoring FeN_4 sites with edge enrichment for boosted oxygen reduction performance in proton exchange membrane fuel cell. *Adv. Energy Mater.* **9**, 1803737 (2019).
6. Zhang, H. *et al.* High-performance fuel cell cathodes exclusively containing atomically dispersed iron active sites. *Energy Environ. Sci.* **12**, 2548–2558 (2019).
7. Wan, X. *et al.* Fe–N–C electrocatalyst with dense active sites and efficient mass transport for high-performance proton exchange membrane fuel cells. *Nat. Catal.* **2**, 259–268 (2019).
8. Li, J. *et al.* Atomically dispersed manganese catalysts for oxygen reduction in proton-exchange membrane fuel cells. *Nat. Catal.* **1**, 935–945 (2018).
9. Wang, X. X. *et al.* Nitrogen-coordinated single cobalt atom catalysts for oxygen reduction in proton exchange membrane fuel cells. *Adv. Mater.* **30**, 1706758 (2018).
10. Shui, J., Chen, C., Grabstanowicz, L., Zhao, D. & Liu, D.-J. Highly efficient nonprecious metal catalyst prepared with metal-organic framework in a continuous carbon nanofibrous network. *Proc. Natl. Acad. Sci.* **112**, 10629–10634 (2015).
11. Proietti, E. *et al.* Iron-based cathode catalyst with enhanced power density in polymer electrolyte membrane fuel cells. *Nat. Commun.* **2**, 416 (2011).
12. Wu, G., More, K. L., Johnston, C. M. & Zelenay, P. High-performance electrocatalysts for oxygen reduction derived from polyaniline, iron, and cobalt. *Science* **332**, 443–447 (2011).
13. Jiao, L. *et al.* Chemical vapour deposition of Fe–N–C oxygen reduction catalysts with full utilization of dense Fe–N_4 sites. *Nat. Mater.* **20**, 1385–1391 (2021).
14. Menga, D. *et al.* Resolving the dilemma of Fe–N–C catalysts by the selective synthesis of tetrapyrrolic active sites via an imprinting strategy. *J. Am. Chem. Soc.* **143**, 18010–18019 (2021).
15. Li, J. *et al.* Thermally driven structure and performance evolution of atomically dispersed FeN_4 sites for oxygen reduction. *Angew. Chem. Int. Ed.* **58**, 18971–18980 (2019).
16. Liu, Q., Liu, X., Zheng, L. & Shui, J. The solid-phase synthesis of an Fe-N-C electrocatalyst for high-power proton-exchange membrane fuel cells. *Angew. Chem. Int. Ed.* **57**, 1204–1208 (2018).
17. Chung, H. T. *et al.* Direct atomic-level insight into the active sites of a high-performance PGM-free ORR catalyst. *Science* **357**, 479–484 (2017).
18. Armel, V. *et al.* Structural descriptors of zeolitic−imidazolate frameworks are keys to the activity of Fe−N−C catalysts. *J. Am. Chem. Soc.* **139**, 453–464 (2016).
19. Zhao, D. *et al.* Highly efficient non-precious metal electrocatalysts prepared from one-pot synthesized zeolitic imidazolate frameworks. *Adv. Mater.* **26**, 1093–1097 (2014).
