# Supplementary material for: Iron atom–cluster interactions increase activity and improve durability in Fe–N–C fuel cells
Source: Nat Commun. 2022 May 26;13:2963. doi: 10.1038/s41467-022-30702-z (PMC9135695; doi:10.1038/s41467-022-30702-z)
Supplement: Supplementary file 3 — Description of Additional Supplementary Files [file 41467_2022_30702_MOESM3_ESM.pdf]

Description of Additional Supplementary Files

File Name: Supplementary Movie 1
Description: MD simulation of Fe–N_4 at 25 °C.

File Name: Supplementary Movie 2
Description: MD simulation of Fe–N_4/Fe_4–N_6 at 25 °C.

File Name: Supplementary Movie 3
Description: MD simulation of Fe–N_4 at 80 °C.

File Name: Supplementary Movie 4
Description: MD simulation of Fe–N_4/Fe_4–N_6 at 80 °C.
